# Sampling time-dependent artifacts in single-cell genomics studies

**DOI:** 10.1186/s13059-020-02032-0

**Published:** 2020-05-11

**Authors:** Ramon Massoni-Badosa, Giovanni Iacono, Catia Moutinho, Marta Kulis, Núria Palau, Domenica Marchese, Javier Rodríguez-Ubreva, Esteban Ballestar, Gustavo Rodriguez-Esteban, Sara Marsal, Marta Aymerich, Dolors Colomer, Elias Campo, Antonio Julià, José Ignacio Martín-Subero, Holger Heyn

**Affiliations:** 1 CNAG-CRG, Centre for Genomic Regulation (CRG), Barcelona Institute of Science and Technology (BIST), Barcelona, Spain; 2 Institute of Biomedical Research August Pi i Sunyer (IDIBAPS), Barcelona, Spain; 3 Rheumatology Research Group, Vall d’ Hebron Research Institute, Barcelona, Spain; 4 Epigenetics and Immune Disease Group, Josep Carreras Research Institute (IJC), Barcelona, Spain; 5 Hematopathology Unit, Hospital Clinic of Barcelona, Barcelona, Spain; 6 Department of Pathology, Medical School, University of Barcelona, Barcelona, Spain; 7 Centro de Investigación Biomédica en Red de Cáncer (CIBERONC), Madrid, Spain; 8 Institució Catalana de Recerca i Estudis Avançats (ICREA), Barcelona, Spain; 9 Universitat Pompeu Fabra (UPF), Barcelona, Spain

**Keywords:** Single-cell, Biobank, RNA sequencing, Peripheral blood mononuclear cells, PBMC, Chronic lymphocytic leukemia, CLL, Sampling, Cryopreservation, Benchmarking

## Abstract

Robust protocols and automation now enable large-scale single-cell RNA and ATAC sequencing experiments and their application on biobank and clinical cohorts. However, technical biases introduced during sample acquisition can hinder solid, reproducible results, and a systematic benchmarking is required before entering large-scale data production. Here, we report the existence and extent of gene expression and chromatin accessibility artifacts introduced during sampling and identify experimental and computational solutions for their prevention.

## Background

Blood cells are an attractive source to systematically identify disease mechanisms and biomarkers, due to its availability in biobanks and large clinical collections. However, although blood samples are generally archived with standardized procedures, upfront sample processing can vary profoundly even within cohorts [1]. In particular, the time between sample extraction and cryopreservation, ranging from hours (local) to days (central) [2], might distort gene expression and epigenetic profiles and could lead to false or biased reporting. Although we have previously demonstrated that cryopreservation is a viable option for single-cell studies [3], the effect of the sampling time on single-cell RNA (scRNA-seq) and ATAC (scATAC-seq) sequencing datasets has not been addressed. However, standardizing sampling conditions is of crucial importance when designing single-cell genomics experiments to avoid technical artifacts in datasets and the misinterpretation of the results. Especially, large-scale consortia with multi-center sampling strategies, such as the Human Cell Atlas project [4] or the single-cell eQTLGen consortium [5], require dedicated standardization efforts to allow an informed decision-making process for guidelines and standards towards high-quality data production. Previous work to determine sampling artifacts in scRNA-seq datasets identified profound alterations of gene expression signatures during sample preparation, storage, and processing, strongly underlining the importance of specific benchmarking efforts [6–9].

In this work, we designed benchmarking experiments to systematically test the effect of varying processing times on single-cell transcriptome and epigenome profiles from healthy and diseased donors, while controlling for technical variability (e.g., batch effects; see the “Methods” section). We isolated peripheral blood mononuclear cells from healthy donors (PBMC) and from patients affected with chronic lymphocytic leukemia (CLL), the most common adult leukemia in the Western world [10]. Samples were either preserved immediately (0 h) or after 2, 4, 6, 8, 24, and 48 h, simulating common scenarios in biobank and clinical routines. Single-cell 3′-transcript counting, full-length transcriptome, and scATAC-seq were performed to monitor gene expression, RNA integrity, and open chromatin variance across preservation time points.

## Results and discussion

**Fig. 1:**
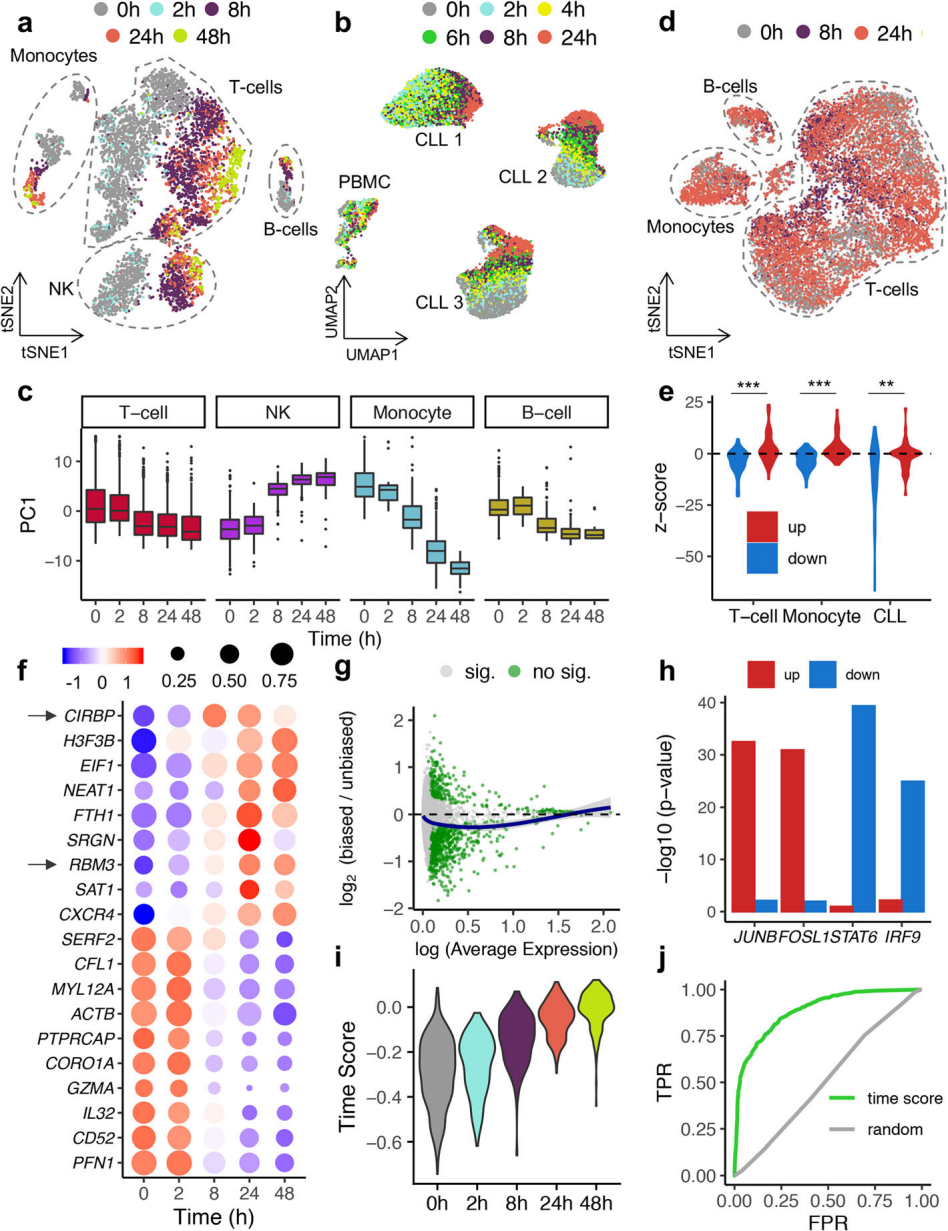
The impact of sampling time on single-cell transcriptional and open chromatin profiles. **a**, **b** scRNA-seq-based tSNE or UMAP embeddings of 7378 PBMC (**a**, male donor) and 22,443 CLL cells (**b**, 3 donors) color-coded by sampling time. **c** Distribution of the first principal component (PC1) across processing times computed for each PBMC subtype independently. **d** scATAC-seq-based UMAP embedding color-coded by sampling time and highlighting major PBMC cell types. Unlabeled cluster corresponds to cells of unknown type. **e** Violin plot showing changes in RNA expression for the 50 genes associated with the top 50 distal (enhancer) peaks changing in accessibility (down: closing sites; up: opening sites); *p* value in *Z* score scale, Wilcoxon test *p < 0.05, **p < 0.01, ***p < 0.001. **f** Dot plot representing the time-dependent expression changes of the top up- and downregulated genes with a minimum log (expression) of 0.5, a minimum absolute log fold-change of 0.2 and an adjusted *p* value < 0.001. The arrows highlight the *cold-inducible response binding protein* (*CIRBP*) and the *RNA Binding Motif Protein 3* (*RBM3*) genes**. g** M (log ratio)-A (mean average) plot showing the log_2_ fold-change between biased (> 2 h) and unbiased (≤ 2 h) PBMC as a function of the log average expression (*Scran* normalized expression values). Significant genes are colored in green (adjusted *p* value < 0.001), and a locally estimated scatterplot smoothing (LOESS) line is drawn in blue. **h** Motif enrichment analysis performed over the DNA sequences of the top 50 distal peaks with a change in accessibility (same peaks as **e**). **i** Time score distribution across processing times (female donor) calculated with the sampling time signature defined in the male PBMC donor. **j** Receiver operating characteristic (ROC) curve displaying the performance of a logistic regression model in classifying “biased” and “unbiased” PBMC

We generated transcriptome and epigenome profiles for 71,064 and 76,146 high-quality cells, respectively. To evaluate the effect of sampling time on single-cell gene expression profiles, we initially obtained fresh PBMC from 2 healthy donors and 3 CLL patients. To simulate local processing, we stored cells prior to cryopreservation at room temperature (RT) for various time intervals up to 8 h. Additionally, we stored cells for 24 h and 48 h, common sampling times for central sample processing. Following scRNA-seq, we detected a marked effect of the sampling time on single-cell transcriptome profiles, initiating after 2 h and increasing in a time-dependent manner (Fig. 1a). This effect was reproducible across all blood cell subtypes from healthy donors and neoplastic cells from CLL patient samples (Fig. 1a,b, Additional file 1: Fig. S1a) and across scRNA-seq technologies (Additional file 1: Fig. S1b). Sampling time correlated with the first principal component (PC1) for all cell subtypes (Fig. 1c), explaining between 15.3% (T cells) and 8.4% (B cells) of the variance contained in the first 50 PC (Additional file 1: Fig. S2a). Moreover, sampling time followed cell type and patient variability as the greatest driver of variance in the PBMC and CLL datasets, respectively (Additional file 1: Fig. S2b-d), and surpassing batch and donor for different cell types (Additional file 1: Fig. S2d-f). Although gene expression profiles varied notably, viable cells did not show signs of reduced RNA integrity across the time points (Additional file 1: Fig. S3).

Contrary to gene expression, prolonged storage at RT did not cause global effects on open chromatin profiles that could be consistently detected across healthy and CLL samples (Fig. 1d, Additional file 1: Fig. S4). However, integrative analysis of scRNA-seq and scATAC-seq data pointed to a deregulation of specific genes through concerted changes at open chromatin sites. Specifically, we detected reduced expression for genes that lose open chromatin sites both at enhancers and promoter sites (Fig. 1e, Additional file 1: Fig. S5).

Next, we aimed to determine the gene signature associated with sampling time interval to characterize, predict, and correct the bias. Therefore, we conducted a differential expression analysis between affected (> 2 h) and unaffected conditions (< 2 h). We detected 1185 differentially expressed genes for PBMC (DEG, 318 up- and 867 downregulated; Fig. 1f,g and Additional file 2: Table 1) and 1868 for CLL samples (378 up- and 1490 downregulated; Additional file 1: Fig. S6a and Additional file 2: Table 1). In addition, we observed a time-dependent decrease in the number of detected genes in both datasets (Additional file 1: Fig. S6b; *p* < 0.001) and a global downregulation of gene expression (Fig. 1g, Additional file 1: Fig. S6a). This global effect has been reported previously in bulk transcriptomics studies [11], pointing to a reduction of the transcriptional rate when cells are removed from their physiological niche (37 °C) and stored at RT (21 °C).

Consistently, Gene Ontology (GO) enrichment analysis revealed a significant increase in the terms “negative regulation of translation” (PBMC) and “negative regulation of transcription by RNA polymerase II” (CLL) as well as a decrease in housekeeping functions such as actin nucleation (Additional file 1: Fig. S6c,d and Additional file 3: Table 2; *p* < 0.001). In line, we detected a pronounced downregulation of immune cell type-specific genes (Fig. 1f, Additional file 1: Fig. S7a and Additional file 2: Table 1) and programs (Additional file 1: Fig. S7b,c and Additional file 3: Table 2) pointing to a loss of identity and function in prolonged storage conditions. Further, two cold-shock master-regulators *Cold Inducible RNA Binding Protein* (*CIRBP*) and the *RNA Binding Motif Protein 3* (*RBM3*) were among the top upregulated genes in both datasets (Fig. 1f and Additional file 1: Fig. S6a). Comparing our profiles with a sampling time-dependent signature detected by bulk gene expression analysis [11] and with tissue-dissociation signature [8], several bona fide stress-regulators were consistently detected across signatures (*NFKBI*, *JUN*, *JUND*, *JUNB*; Additional file 1: Fig. S8a,b). However, there was a marginal global intersection (Additional file 1: Fig. S8a,c and Additional file 4: Table 3), which highlights the need for technology-specific benchmarking efforts of technical confounders. As an example, gene markers for splicing events detected in bulk analysis [12] were undetectable with scRNA-seq, while *CIRBP* and *RBM3* were only found in single-cell experiments.

Motif enrichment analysis at sampling time-sensitive enhancers identified by scATAC-seq pointed to a significant increase in the accessibility of transcription factor binding sites (TFBS) of early stress response genes, such as *JUNB* and *FOSL1* (Fig. 1h and Additional file 5: Table 4), as previously shown in scRNA-seq studies [8]. Further, we detected a significant decrease in accessibility at TFBS of immune and inflammation-related genes, such as *STAT6* and *IRF9* (Fig. 1h, Additional file 5: Table 4), in line with the downregulation of immune response genes at the transcript level.

**Fig. 2:**
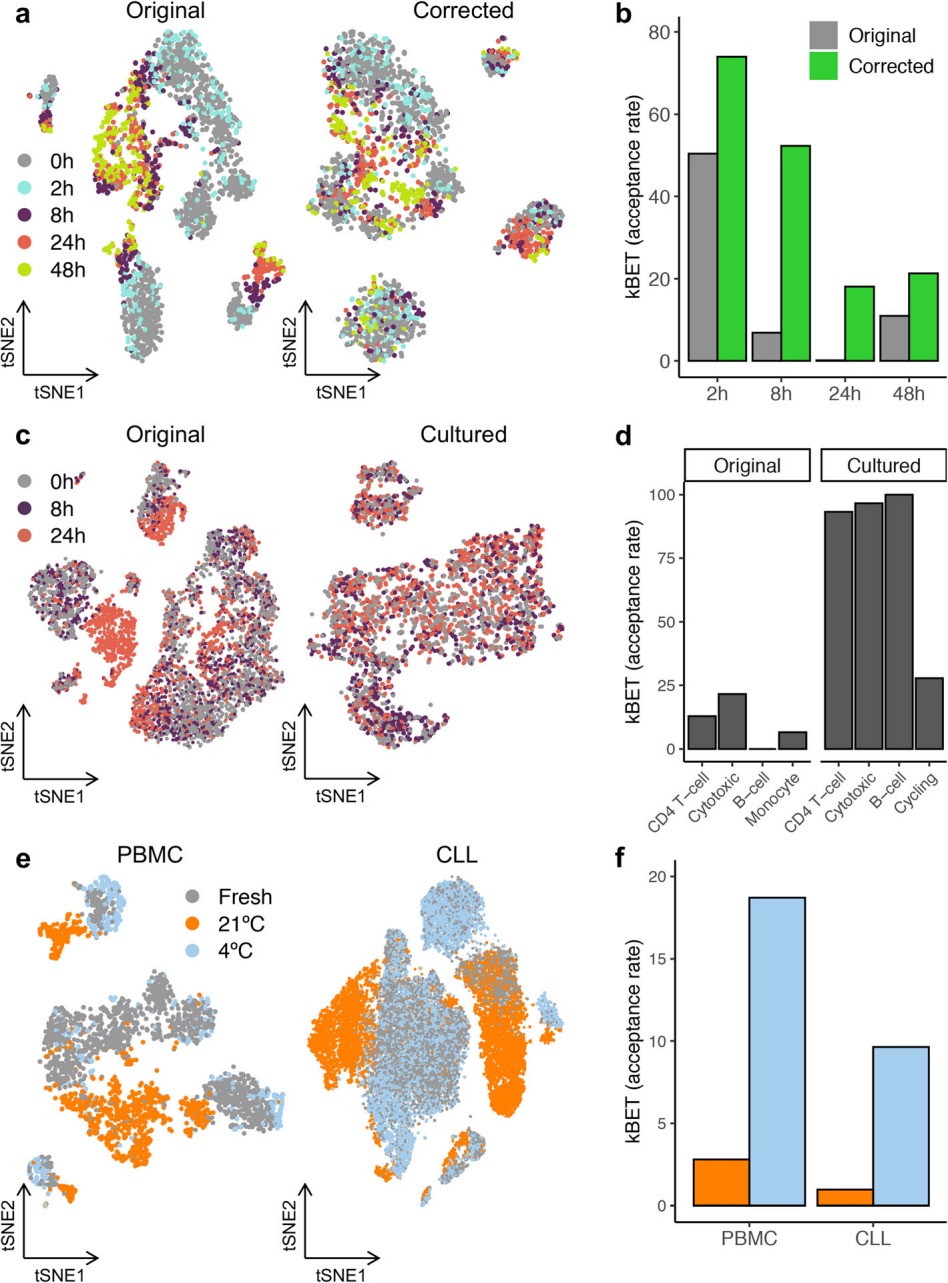
Solutions to correct or prevent sampling time-induced artifacts. **a** tSNEs displaying the effect of varying processing times on the transcriptome profiles of 7378 PBMC before (left) and after (right) regressing out the time score for every highly variable gene. **b** kBET acceptance score distribution across sampling times with or without the computational correction. **c** tSNE showing the effect of PBMC culturing and activation with anti-CD3 Dynabeads over 2 days. **d** kBET acceptance score distribution across cell types with or without cell culture/activation. **e** tSNE highlighting the sampling effect between cells cryopreserved immediately (fresh, 0 h) or after 24 h and 48 h stored cold (4 °C) or at RT (21 °C). **f** kBET acceptance score distribution across storage temperatures

We next sought to identify solutions for retrospective study designs and prospective cohort collection. To predict such sampling time effect, we calculated a time score using the abovementioned signature [13], which classified cells to be affected by sampling time (AUC = 0.888, Fig. 1i, j). In silico data correction is commonly applied to diminish the effects of technical or biological variability in scRNA-seq datasets by scoring and regressing out specific gene sets [14]. Applying such strategy on the time gene expression score, we were able to reduce the sampling effect, especially for samples with local processing (< 8 h). This correction was robust for different PBMC subtypes (Kbet score [15]; Fig. 2a,b) and neoplastic cells from CLL patients (Additional file 1: Fig. S9a) as well as simulated datasets with varying proportions of affected cells (Additional file 1: Fig. S9b), suggesting a broad application spectrum. Importantly, the correction conserved biological variance related to cell identity in blood and inter-individual variation in CLL patients. However, owing to the Simpson’s paradox [16] and to gene expression pleiotropy [17], regressing out technical confounders can remove subtle biological heterogeneity and homogenize cell subpopulations, which can challenge data interpretation. Consequently, we sought experimental alternatives to reduce sampling effects in retrospective study designs. We reasoned that the magnitude of gene expression alterations could be diminished by cell culture and through the activation of cell type-specific programs. Hence, we utilized PBMC (cryopreserved at 0/8/24 h) and processed them directly (day 0) or after 2 days in cell culture with simultaneous T cell activation (anti-CD3, day 2). Strikingly, the culturing/activation reduced the sampling induced artifact, quantifiable through increased similarities between the time points (Kbet score; Fig. 2c, d). In line, after cell culture, no significant differences in time score could be observed between the time points (Additional file 1: Fig. S10). It is of note that culturing and activation result in specific gene expression profiles that allow the simulation of disease phenotypes (e.g., auto-immune diseases), but might also distort expression profiles observed in vivo (Additional file 1: Fig. S11 and Additional file 6: Table 5).

Finally, we hypothesized that cold sample storage could prevent time-related sampling effects by minimizing active and passive cell responses. In line, tissues (lung, pancreas, and esophagus) preserved at cold temperatures (4 °C) did not show altered single-cell gene expression profiles or cell type composition changes up to 72 h of storage [18]. Importantly, changing storage temperatures could be readily implemented in prospective cohort study designs to enable subsequent scRNA-seq. Indeed, when PBMC or CLL samples were stored at 4 °C until cryopreservation (24 and 48 h for PBMC; 2, 4, 6, 8, and 24 h for CLL), we did not detect global gene expression artifacts, an effect observed for both healthy and CLL samples and replicated across donors and technologies (Fig. 2e,f and Additional file 1: Fig. S12a,b). Although we detected an upregulation of stress-regulators (*NFKBIA*, *FOS*, *JUN*, *JUNB*) in PBMC stored > 24 h at 4 °C (Additional file 1: Fig. S12c), we identified between 254 (2 h) and 362 (24 h) DEG in CLL cells, notably less than when kept at RT (between 797 and 1956 DEG at 2 h and 24 h, respectively; Additional file 1: Fig. S12d).

## Conclusions

We report that varying sampling times until cryopreservation is a driver of technical variability in scRNA-seq and scATAC-seq profiles. This bias was ubiquitous across cell types, donors, protocols, and disease status, thus likely presenting a highly frequent obstacle in transcriptome and epigenome cohort studies. Despite the substantial impact on single-cell datasets, the proposed computational corrections, cell culture, and storage adjustments allow the design of reliable retro- or prospective studies of immune cells from archived sample collections. However, sampling effects can be tissue- and cell type-specific; thus, dedicated benchmarking efforts are required for sample types other than the ones tested here. In general, sampling artifacts are important to consider when planning single-cell experiments. Failing to select suitable samples or to correct datasets will lead to biased or false reporting.

## Methods

### PBMC isolation and cryopreservation

Peripheral venous blood samples were collected by venipuncture from two voluntary blood donors, one male and one female. Blood samples were collected in ACD-tubes and stored at room temperature (RT) or 4 °C. In the former condition, peripheral blood mononuclear cells (PBMC) were isolated at 2, 8, 24, and 48 h. For samples stored at 4 °C, PBMC were isolated at 24 and 48 h. PBMC separation was performed using Ficoll density gradient centrifugation. For each condition, 12 ml of blood was diluted with an equal volume of pre-warmed RPMI 1640 culture medium (Lonza). The diluted blood was then carefully layered onto a Leucosep tube (Greiner Bio-One) prefilled with 15 ml of Ficoll-Plus (GE Healthcare Biosciences AB) and centrifuged for 15 min at 800×*g* and RT (without acceleration and brake). After centrifugation, PBMC were collected with a sterile Pasteur pipette into a 50-ml tube, diluted up to 10 ml with pre-warmed RPMI medium and centrifuged for 10 min, at 400*×g* and RT. Following a second washing step with 5 ml of RPMI medium and a 5-min centrifugation, PBMC were resuspended in 8 aliquots of freezing media. Freezing media consisted of RPMI 1640 with 20% heat-inactivated fetal bovine serum (Sigma-Aldrich), 10% DMSO (Sigma-Aldrich), and penicillin-streptomycin 1:1000 (Lonza). One-milliliter aliquots, with approximately 1 × 10e6 cells/ml, were gradually frozen using a commercial freezing box (Mr. Frosty, Nalgene) at − 80 °C for 24 h and then stored in a vapor-phase liquid nitrogen tank at − 150 °C.

Cryopreserved (− 80 °C) PBMC samples were rapidly thawed in a 37 °C water bath. Each sample was transferred into a 15-ml Falcon using a 1000-μl cut tip without mixing by pipetting. Next, 1 ml of 37 °C pre-warmed media (Hibernate-A supplemented with 10% FCS; ThermoFisher) was added dropwise with gentle swirling of the sample. After 1-min incubation, 2 ml of pre-warmed media was added and incubated for 1 min. Next, 5-ml pre-warmed media was gently added, inverted, and incubated (1 min). This step was repeated once. Finally, the samples were centrifuged at 700×*g* for 5 min (4 °C). The supernatant was removed, and the pellets re-suspended in 100 μl of Cell Staining Buffer (BioLegend). Of note, we did not observe an increase of damaged/dead cells (cell viability staining) towards the later time points with an average viability of 95% (range, 88–98%) across time points. An increased fraction of debris could be observed at 24 and 48 h, which, however, did not result in reduced data quality (e.g., proportion of excluded cells) during data analysis (Additional file 7: Table 6).

CLL patient samples were obtained from freshly extracted blood, stored either at RT or 4 °C. Mononuclear cells were isolated after Ficoll density gradient centrifugation, at 2, 4, 6, 8, and 24 h after patient blood extraction. The cells were directly cryopreserved with freezing media (RPMI 1640 with 20% FBS and 10% DMSO), in the concentration of 5–10 × 10e6 cells/ml, according to standardized protocol. The tumor cell content of all the samples was > 80%, as assessed by immunostaining of CD19, CD20, CD5, and CD45 followed by flow cytometry. All patients gave informed consent for their participation in the study according to International Cancer Genome Consortium (ICGC) guidelines.

Cell hashing was performed following the manufacturer’s instructions (Cell hashing and Single Cell Proteogenomics Protocol Using TotalSeq™ Antibodies; BioLegend). Therefore, samples were incubated 10 min at 4 °C with Human TruStain FcX™ Fc Blocking reagent (BioLegend). Next, sample-specific TotalSeq antibodies (anti-human Hashtag 1-8, Biolegend) were added with subsequent incubation on ice for 45 min. Cells were washed once with cold 1X PBS supplemented with 0.0005% BSA (ThermoFisher) and pelleted at 700×*g* for 5 min. A single-cell solution was obtained resuspending the pellet in 1X PBS (0.0005% BSA) and filtering it through a 40-μm cell strainer. The cells were counted in an automatic cell counter (Countess® v.2, ThermoFisher).

### PBMC isolation and activation

Peripheral venous blood samples were collected by venipuncture from one voluntary donor (male). Blood samples were collected in 10-ml Vacutech Vacuum Blood Collection Tubes K2/K3 EDTA (Becton Dickinson) and stored at RT. Peripheral blood mononuclear cells (PBMC) were isolated at 0, 8, and 24 h after blood collection. PBMC separation was performed using Ficoll density gradient centrifugation. For each condition, 9 ml of blood was diluted with an equal volume of 1X PBS (Gibco). The diluted blood was then carefully layered onto 9 ml of Lymphoprep solution (STEMCELL Technology) and centrifuged for 15 min at 700×*g* and RT (without acceleration and brake). After centrifugation, PBMC were collected and washed twice with 10 ml of 1X PBS. The pellet was resuspended with 10 ml of 1X PBS, and cells were counted with a TC20™ Automated Cell Counter (Bio-Rad Laboratories). PBMC were again centrifuged for 5 min at 700×*g* and resuspended in an appropriate volume of freezing media (RPMI with 10% heat-inactivated fetal bovine serum and 10% DMSO). Aliquots of ~ 0.5 × 10e6 cells/ml were gradually frozen using a commercial freezing box (Mr. Frosty, Nalgene) at − 80 °C for 24 h and then stored in a vapor-phase liquid nitrogen tank at − 150 °C.

For T cell activation, cells were thawed in MACS buffer (1X PBS, 4% FBS, 2 mM EDTA), centrifuged during 5 min at 700×*g* and RT, and resuspended in pre-warmed culture media (RPMI, 1% Pyruvate, 20% FBS, Pen/Strep, DNase 100 U/ml). A TC20™ automated cell counter was used to assess cell number and viability. The number of only viable cells was used to calculate volumes for cell seeding. For each condition, 200,000 live cells were seeded into two wells of a 96-well round bottom plate (Sigma Aldrich) for a total of 400,000 cells per condition (time point). Dynabeads Human T-Activator CD3/CD28 (Thermo Fisher Scientific) were transferred to a 1.5-ml tube (5 μl/well), washed twice with 1 ml of cell culture media, and resuspended with 10 volumes of cell culture media. Fifty microliters of resuspended beads was added to each well for T cell activation and expansion. Cells were incubated during 24 h at 37 °C with 5% CO_2_ and 5% humidity. The remaining cells (~ 350,000 cells per condition) were used as a control (day 0) for T cell activation. Cells subjected to T cell activation protocol were collected in a 1.5-ml tube and stained with DAPI (Thermo Fisher Scientific) at 1 μM final concentration. DAPI-negative live individual cells were sorted with a BD FACSAria™ Fusion Flow cytometer (BD Biosciences) in 1X PBS supplemented with 0.05% BSA.

Samples subjected to T cell activation treatment, as well as corresponding control samples, were subjected to a Cell Hashing protocol before proceeding to scRNA-seq. Cell hashing was performed following the manufacturer’s instructions (Cell hashing and Single Cell Proteogenomics Protocol Using TotalSeq™ Antibodies; BioLegend). Cells were counted with a TC20™ Automated Cell Counter, and an equal number of cells was taken for each condition. Briefly, samples were resuspended in Cell Staining Buffer (BioLegend), incubated 10 min at 4 °C with Human TruStain FcX™ Fc Blocking reagent (Bio Legend). To each condition, a specific TotalSeq-A antibody-oligo conjugate (anti-human Hashtag 1-8, Biolegend) was added and incubated on ice for 1 h. Cells were then washed three times with cold PBS-0.05% BSA (ThermoFisher) and centrifuged for 5 min at 700×*g* at 4 °C. Finally, cells were resuspended in an appropriate volume of 1X PBS-0.05% BSA in order to obtain a final cell concentration > 500 cells/ul, suitable for 10x Genomics scRNA-seq. An equal volume of hashed cell suspension from each of the conditions (0 h, 8 h, and 24 h) was mixed and filtered with a 40-μm strainer. Cell concentration was verified by counting with a TC20™ Automated Cell Counter.

### Single-cell RNA sequencing

Cells were partitioned into Gel Bead In Emulsions with a Target Cell Recovery of 10,000 total cells. Sequencing libraries were prepared using the single-cell 3′ mRNA kit (V2 for PBMC samples and V3 for CLL and cultured PBMC samples; 10x Genomics) with some adaptations for cell hashing, as indicated in TotalSeq™-A Antibodies and Cell Hashing with 10x Single Cell 3′ Reagent Kit v3 3.1 Protocol by BioLegend. Briefly, 1 μl of 0.2 μM HTO primer (Hashtag Oligonucleotides; GTGACTGGAGTTCAGACGTGTGC*T*C; *Phosphorothioate bond) was added to the cDNA amplification reaction in order to amplify the hashtag oligos together with the full-length cDNAs. A SPRI selection cleanup was done in order to separate mRNA-derived cDNA (> 300 bp) from antibody-oligo-derived cDNA (< 180 bp), as described in the abovementioned protocol. 10x cDNA sequencing libraries were prepared following 10x Genomics Single Cell 3′ mRNA kit protocol, while HTO cDNAs were indexed by PCR as follows. Briefly, 5 μl of purified hashtag oligo cDNA was mixed with 2.5 μl of 10 μM Illumina TruSeq D70X_s primer (IDT) carrying a different i7 index for each sample, 2.5 μl of SI primer from 10x Genomics Single Cell 3′ mRNA kit, 50 μl of 2X KAPA HiFi PCR Master Mix (KAPA Biosystem), and 40 μl of nuclease-free water. The reaction was carried out using the following thermal cycling conditions: 98 °C for 2 min (initial denaturation), 12 cycles of 98 °C for 20 s, 64 °C for 30 s, 72 °C for 20 s, and a final extension at 72 °C for 5 min. The HTO libraries were purified with 1.2 X SPRI bead selection. Size distribution and concentration of cDNA and HTO libraries were verified on an Agilent Bioanalyzer High Sensitivity chip (Agilent Technologies). Finally, sequencing of HTO and cDNA libraries was carried out on a HiSeq4000 or NovaSeq6000 system (Illumina).

### Single-cell ATAC sequencing

For the single-cell ATAC-seq experiments, we analyzed one PBMC and one CLL sample isolated after 0 h, 8 h, and 24 h of blood storage at room temperature before cryopreservation. Frozen samples were rapidly thawed in a 37 °C water bath. Each sample was transferred into a 15-ml Falcon using a 1000-μl cut tip without mixing by pipetting. Next, 1 ml of 37 °C pre-warmed media (Hibernate-A supplemented with 10% FCS) was added dropwise with gentle swirling of the sample. After 1 min of RT incubation, additional 2 ml of pre-warmed media was added. The samples were again kept at RT for 1 min, before 5 ml of pre-warmed media was gently added. This step was repeated once. Then, samples were centrifuged at 700×*g* for 5 min. Supernatant was removed and the pellets resuspended in 500 μl of PBS supplemented with 0.05% BSA. Cell concentration and viability were determined with a TC20™ Automated Cell Counter.

Nuclei isolation was performed following the “Nuclei Isolation for Single Cell ATAC Sequencing demonstrated protocol” (10x Genomics). Briefly, 1,000,000 cells from the CLL sample and 300,000 cells from PBMC were transferred to a 1.5-ml microcentrifuge tube and centrifuged at 500×*g* for 5 min at 4 °C. The supernatant was removed without disrupting the cell pellet, and 100 μl of chilled Lysis Buffer (10 mM Tris-HCl (pH 7.4); 10 mM NaCl; 3 MgCl2; 0.1% Tween-20; 0.1% Nonidet P40 Substitute; 0.01% Digitonin and 1% BSA) was added and pipette-mixed 10 times. Samples were then incubated on ice during 3 min. Following lysis, 1 mL of chilled Wash Buffer (10 mM Tris-HCl (pH 7.4); 10 mM NaCl; 3 MgCl2; 0.1% Tween-20 and 1% BSA) was added and pipette-mixed. Nuclei were centrifuged at 500×*g* for 5 min at 4 °C, and the supernatant removed without disrupting the pellet. Based on the starting number of cells and assuming a 50% loss during the procedure, nuclei were resuspended into the appropriate volume of chilled Diluted Nuclei Buffer (10x Genomics) in order to achieve a nuclei concentration of 1540–3850 nuclei/μl, suitable for a Target Nuclei Recovery of 5000. The resulting nuclei concentration was determined using a with a TC20™ Automated Cell Counter.

scATAC-seq libraries were prepared according to the Chromium Single Cell ATAC Reagent Kits User Guide (10x Genomics; CG000168 Rev. B). Briefly, the transposition reaction was prepared by mixing the desired number of nuclei with ATAC Buffer (10X Genomics) and ATAC Enzyme (10X Genomics), before incubation for 60 min at 37 °C. Nuclei were partitioned into Gel Bead-In-Emulsions (GEMs) by loading the following into a Chip E: the master mix (previously added to the same tube of the transposed nuclei), the Chromium Single Cell ATAC Gel Beads (10X Genomics), and the Partitioning Oil (10X Genomics). After the run into the Chromium Controller, the DNA linear amplification was performed by incubating the GEMs at the following thermal cycling conditions: 72 °C for 5 min, 98 °C for 30 s, 12 cycles of 98 °C for 10 s, 59 °C for 30 s, and 72 °C for 1 min. GEMs were broken using the Recovery Agent (10X Genomics), and the resulting DNA was purified by Dynabeads and SPRIselect reagent (Beckman Coulter; B23318) bead cleanups. Indexed sequencing libraries were obtained by mixing the amplification product with the Sample Index PCR Mix (10X Genomics) and the Chromium i7 Sample Index (10x Genomics) and incubating at the following thermal cycling conditions: 98 °C for 45 s, 12 cycles of 98 °C for 20 s, 67 °C for 30 s, 72 °C for 20 s, and with a final extension of 72 °C for 1 min. Sequencing libraries were subjected to a final bead cleanup SPRIselect reagent and quantified on an Agilent Bioanalyzer High Sensitivity chip (Agilent Technologies). Finally, libraries were loaded on an Illumina HiSeq 2500 system in Rapid Run mode using the following read length: 50 bp Read 1N, 8 bp i7 Index, 16 bp i5 Index, and 50 bp Read 2N.

### Primary processing and demultiplexing

We processed sequencing reads with CellRanger v3.0.0 for the PBMC data and v3.0.2 for the CLL and T cell activation data. We used the human GRCh38 assembly as reference genome. To specify the hashtag oligonucleotide (HTO) libraries, the cDNA libraries, and the HTO sequences, we followed the “Feature Barcoding Analysis” pipeline, available at https://support.10xgenomics.com/single-cell-gene-expression/software/pipelines/latest/using/feature-bc-analysis. We set the --chemistry and --expect-cells flags of *cellranger count* to “SC3Pv3” and “5000”, respectively. We demultiplexed cell hashtags as described in Stoeckius et al. [19] for each batch and donor separately. Briefly, we normalized hashtag oligonucleotide (HTO) counts using a centered log ratio (CLR), in which each count is divided by the geometric mean of a HTO across cells and log-transformed. We then clustered barcodes using *k*-medoids with *k* equal to the number of conditions (*k* = 4 for batch 03, and *k* = 8 for batch 04), which allowed us to identify the background distribution of each HTO. We re-clustered Male 04 with *k* = 3, as no clear signal for the HTO “24 h 4 °C” was detected. Subsequently, we considered the top 0.5% normalized HTO counts of the background distribution as outliers and excluded them. We classified barcodes to a given condition if the normalized HTO counts of that condition exceeded the 0.99 quantile. We discarded both barcodes that were assigned to more than one condition (multiplets) and barcodes that were not assigned to any condition (negatives). In subsequent datasets (CLL and T-cell activation), we demultiplexed the HTO with Seurat’s built-in functions [20]. Of note, all sampling time point showed comparable dataset qualities (e.g., library size and mitochondrial read content; Additional file 7: Table S6).

### Quality control and normalization

We performed quality control (QC) and normalization separately for each dataset (PBMC, CLL, T-cell activation). Following the guidelines from Luecken et al. [21], we inspected the distributions of three main QC metrics: library size (total UMI), library complexity (number of detected genes), and mitochondrial expression. Importantly, we analyzed these metrics jointly to ensure that cells with high mitochondrial expression were not metabolically active. Finally, we analyzed one of the CLL donors independently as it showed markedly different distributions in QC metrics. We classified as damaged cells those barcodes with an aberrantly low number of UMI and genes, or with an abnormally high mitochondrial expression. Likewise, we classified as doublets those barcodes that possessed and aberrantly large library size and complexity. We also leveraged DoubletFinder [22] to detect doublets that shared the same HTO and were not outliers in any qc metric. We ruled out genes that were detected in less than 10 (CLL) or 15 cells (T cell activation). Finally, we used the *Scran* 1.10.2 package [23] to normalize UMI counts with cell-based size factors. We provide a supplementary table with the number of cells before and after QC, the average library size, library complexity, and mitochondrial fraction stratified by time, donor, and temperature (Additional file 7: Table 6).

### Cell type annotation

Cell type annotation was performed within the *Seurat* framework [24] (Additional file 1: Fig. S13). To cluster cells, we (i) identified overdispersed genes with the *FindVariableFeatures* function (using default parameters), (ii) scaled UMI counts and regressed out the batch effect, (iii) performed principal component analysis (PCA), (iv) used these PCs to create a *k*-nearest neighbors graph with the *FindNeighbors* function, and (v) clustered cells with the *FindClusters* function. We set the resolution parameter to 0.05, 0.01, and 0.1 for the PBMC, CLL, and T cell activation data, respectively. Finally, we used well-known marker genes to annotate each cluster to its specific cell type. We provide a supplementary table with the number of cells per cell type stratified by time, donor, and temperature (Additional file 7: Table 6).

### Variance analysis

To quantify and compare sampling time with other sources of variance, we followed two complementary approaches. *First*, we defined *ρ*_p_ as a universal measure of cell similarity, as Skinnider et al. [25] described this proportionality metric as the most accurate to measure cell-cell association. We then downsampled T cells and B cells to 50 cells per sampling time to ensure that different cell types and time points were equally represented. Likewise, we downsampled each cluster of leukemic cells (one per donor) in a similar manner. We obtained a distance matrix by computing all pairwise *ρ*_p_ as described in Skinnider et al. Finally, we sorted the distance matrix by cell type and time to allow for interpretation.

Second, we downsampled monocytes and NK cells as described before and merged it with the T and B cell dataset. We then regressed the *scran*-normalized gene expression values of 5282 genes (union of highly variable features of the merged and cell type-specific datasets) on one of four explanatory variables (cell type, time, donor, and batch), and extracted a distribution of *r*^2^ values for each variable. To conduct a similar analysis in the Smart-seq2 dataset (T cells), we used the *plotExplanatoryVariables* function from *Scater* [26].

### Differential expression analysis (DEA)

To find the scRNA-seq signature, we divided cells in the PBMC and CLL datasets in time-biased (*t* > 2 h) and time-unbiased (*t* < =2 h). Subsequently, we performed a Wilcoxon signed-rank test to test for differential expression for each gene. Soneson et al. [27] reported that this test is among the best performing for scRNA-seq DEA analysis. Vieth et al. [28] showed that with *Scran* normalization, there is no need for scRNA-seq-tailored statistical tests. To define the sampling time signatures, we performed the same approach but setting the logfc.threshold of Seurat’s *FindMarkers* function to logfc.threshold = 0.25 to increase the specificity.

### Gene Ontology (GO) enrichment analysis

To elucidate biological processes affected by sampling time, we conducted a GO enrichment analysis with the *GOstats* 2.48.0 package [29]. We used as target set the entrez identifiers of the upregulated (log fold-change > 0) or downregulated (log fold-change < 0) genes in the sampling time signature, and as universe set the entrez identifiers of all genes that we included in the analysis. Finally, we filtered out GO terms that were too general (size ≥ 600), or too specific (size < 3). In addition, we only retained GO terms with a *p* value lower than 0.05 and an odds ratio greater than 2.

### Integration of the sampling signatures

To compare the sampling time signatures detected for PBMC and CLL with other condition-specific signatures, we downloaded the DEG tables for the studies by Baechler et al. [11] and van den Brink et al. [8]. We defined as signature those genes with an adjusted *p* value < 0.001. Furthermore, we subsetted the Baechler signature to the top 250 genes to harmonize the number of DEG across signatures. Finally, we calculated a signature-specific score using the *AddModuleScore* function from Seurat.

### Prediction of sampling time-biased cells

To predict sampling time-biased cells, we used the *AddModuleScore* function of Seurat to compute a time score per cell using a signature calculated on the male donor (training set). We then fitted a logistic regression model using the time score as explanatory variable. Subsequently, we predicted the probability of being “biased” for every cell of the female donor (test set) and found the area under the curve (AUC) with the “AUC” function from the cvAUC v1.1.0 package. To test the significance of our signature, we repeated the process with a signature defined on random genes.

### Computational correction sampling time signature

To correct the time-biased transcriptomes in the female PBMC dataset (test set), we regressed the expression of each gene on the time score and kept the scaled and centered residuals as the variance in gene expression not explained by time. We performed this process for each cell type independently to minimize Simpson’s paradox.

As all the analysis above used a similar proportion of “biased” and “unbiased” cells, we sought to test the effect of varying percentages of biased cells on the time score computation and regression. In this setting, we performed bootstrapping as follows: first, we sampled 300 cells with replacement from the Smart-seq2 dataset, enforcing an approximate percentage of time-affected cells. Second, we computed the average Silhouette width between affected and unaffected cells. Finally, we computationally corrected the transcriptome profiles and recalculated the average Silhouette width. We repeated this process 25 times for each set of percentages ranging from 10 to 90% of affected cells.

### *k*-nearest-neighbor batch-effect test (kBET)

To assess the mixability between cells of different time points in the presence or absence of our corrections, we used the kBET metric [15]. Briefly, kBET compares the label distribution of the local *k*-nearest neighborhood of a given cell with the global distribution with a Pearson’s *χ*^2^ test, with the null hypothesis that, if samples are well-mixed, both distributions are equal. We ran kBET with the cells embedded in UMAP space and with default parameters. We defined the acceptance rate as the percentage of tested cells with a *p* value > 0.05, and as rejection rate 100-acceptance rate.

### Smart-seq2 validation

To confirm that the results obtained from 10x Genomics-derived data were technology-independent, we profiled the transcriptome of 376 CD3+ T cells with Smart-seq2 [30]. The cells originated from the same donors as in the 10x Genomics experiments and were distributed across four 96-well plates (all time points per plate). We discarded 60 cells that either had < 75,000 or > 1,000,000 total counts, < 435 detected genes or a mitochondrial expression > 20%. Similarly, we filtered out 6542 genes that had an average expression across cells < 1. We normalized gene counts with the *Scran* package, which removed the batch effect between plates. Finally, we clustered cells with the *SC3* 1.10.1 package [31], as it outperforms other tools for small datasets [32].

### ATAC-seq data analysis

ATAC-seq data from 10x Genomics was processed with CellRanger-atac 1.1.0. Differential accessibility to detect changes in open chromatin sites was performed with Wilcoxon-Mann-Whitney rank sum test for high-throughput expression profiling data (R BioQC v1.0.0). Motif enrichment analysis was performed using the package *motifcounter* v.1.10.0 [33] with default parameters, and the motifs were downloaded from JASPAR database (579 motifs from JASPAR CORE VERTEBRATES, http,//jaspar.genereg.net/downloads/). The background distribution was computed over the total peaks called in the datasets (56,627 in the PBMC and 80,861 in the CLL). We provide a supplementary table with multiple QC metrics (number of high-quality cells and detected peaks, among others) stratified by experiment, donor, and time (Additional file 7: Table 6).

## Supplementary information

**Additional file 1: Supplementary material.** Supplementary Figs. 1–13 and Supplementary figure and table legends.

**Additional file 2: Table 1.** Differentially expressed genes in prolonged (> 2 h) storage of PBMC, CLL, T-cells, NK, monocytes and B-cells.

**Additional file 3: Table 2.** Enriched Gene Ontology (GO) terms in storage time-dependent DEG in PBMC, CLL, T-cells, NK, monocytes and B-cells.

**Additional file 4: Table 3.** Gene signature associated to storage time in PBMC (measured by scRNA-seq and microarray), CLL (measured by scRNA-seq), and collagenase-dependent tissue dissociation (van den Brink).

**Additional file 5: Table 4.** Transcription factor binding site motif enrichment analysis at sampling time-sensitive enhancers.

**Additional file 6: Table 5.** Differentially expressed genes in cultured CD4+ T-cells, cytotoxic cells and B-cells activated with anti-CD3 antibody.

**Additional file 7: Table 6.** scRNA-seq and scATAC-seq quality control metrics stratified by time, donor and temperature in both experiments (PBMC and CLL).

**Additional file 8.** Review history.

## Data Availability

The complete raw data (fastqs) and feature-barcode matrices are available at the Gene Expression Omnibus (GEO) under GSE132065. https://www.ncbi.nlm.nih.gov/geo/query/acc.cgi?acc=GSE132065 [34]. The code and analysis notebooks to reproduce the aforementioned analysis are hosted at https://github.com/massonix/sampling_artifacts [35].
