# Supplementary material for: Sampling time-dependent artifacts in single-cell genomics studies
Source: Genome Biol. 2020 May 11;21:112. doi: 10.1186/s13059-020-02032-0 (PMC7212672; doi:10.1186/s13059-020-02032-0)

Additional Figures

Additional file 1: Figure S1. The impact of sampling time on single-cell transcriptional profiles. (a,b) tSNE embedding of **(a)** 2,460 PBMC (10x Genomics, female donor) and **(b)** 198 CD3-positive cells (Smart-seq2) colored by sampling time.

Additional file 1: Figure S2. Drivers of variance in PBMC and CLL scRNA-seq datasets. (a) Percentage of the variance explained by the first 50 principal components (PC) for each PC computed for each cell type independently. **(b, c)** Distance matrix of down-sampled T- and B-cells (b) and neoplastic CLL cells (c) obtained by computed all pairwise ρ_p values. **(d, e)** Distribution of r^2 obtained by regressing the expression of selected genes in the 10X PBMC (d) and Smart-seq2 T-cell datasets (e) onto different explanatory variables. **(f)** Same as (d) but conducted for each cell type independently.

Additional file 1: Figure S3. Conserved RNA integrity across sampling time points. Mapping distribution of sequencing reads from 5` to 3` for full-length single-cell library preparation from 198 CD3-positive cells (Smart-seq2) across different time-points. Each line represents the distribution of a single cell. The total number of cells per time point is indicated.

Additional file 1: Figure S4. Single-cell ATAC-seq data analysis. (**a,b**) Distribution of peak heights in the different conditions. Peak heights were estimated by summing the reads of all the cells of a given condition and dividing by the peak width (bp). The CLL 24 h sample displays an abundance of small peaks compared to 0 and 8 hours, an effect not observed PBMC datasets, pointing to be a technical rather than sampling bias. (**c,d**) Distribution of peak widths in the different conditions. CLL 24 h sample had narrower peaks (visible especially in the highlighted range). (**e,f**) Relationship between total sequencing reads and duplicated reads at single-cell resolution. Duplicated reads originate from PCR amplification of the same amplicon. A lower amount of duplicated reads at a given sequencing depth, as observed in the CLL 24 h sample, indicates higher background signal. (**g,h**) Relationship between unique sequencing reads (ie, after removal of duplicate reads) and detected open regions (ie peaks) at single-cell level. The CLL 24 h sample had less detected open regions for the same amount of unique reads, again compatible with a higher background noise. (**i**) t-SNE plots of scATAC-seq dataset of PBMC showing the expression of CD3G (T-cells), NKG7 (Natural killer cells), MS4A1 (B-cells) and IL1B (Monocytes).

Additional file 1: Figure S5. Violin plots showing the changes in RNA expression for the 50 genes associated with the top 50 promoter peaks with a change in accessibility (UP and DOWN, Wilcoxon test p-value in Z-score scale; *p<0.05, **p<0.01).

Additional file 1: Figure S6. Sampling time scRNA-seq signature. (**a**) M(log ratio)-A(mean average) plot showing the $\log_2$ fold-change between biased (>2h) and unbiased (<=2h) CLL cells as a function of the log average expression (*Scran* normalized expression values). Significant genes with an adjusted p-value <0.001, an absolute log2 fold-change >0.25 and a log (average expression) >0.5 are colored in green. Locally estimated scatterplot smoothing (LOESS) line is drawn in blue. (**b**) Distribution of the number of detected genes across processing types (fresh, local, central) in both PBMC and CLL cells. *p<0.05, **p<0.01, ***p<0.001. (**c**,**d**) Gene ontology enrichment analysis of the up- and down-regulated genes in the sampling time signatures in PBMC (**c**) and CLL (**d**) cells.

Additional file 1: Figure S7. Sampling time induces a loss of cell identity in PBMC. (**a**) Heatmap showing the cell type specificity of the sampling time transcriptome signature (top 100 differentially expressed genes per cell type). (**b**) Time-dependent loss of cell identity for each PBMC subtype. Cell identity scores are calculated using manually curated cell subtype markers. (**c**) Gene ontology enrichment analysis of the down-regulated genes in each cell type-specific sampling time signature.

Additional file 1: Figure S8. Condition-specific gene expression signatures comparison. (a) Overlap between the sampling time scRNA-seq signatures for PBMC and CLL signatures described in this manuscript, the collagenase-dependent tissue dissociation scRNA-seq signature (van den Brink) and the sampling time RNA microarray signature (Baechler). **(b)** Heatmap showing the genes present in more than one signature, discarding those that only intersect in our PBMC and CLL signatures. Red and blue indicate up- and down-regulation, respectively. **(c)** tSNE embedding of 9,803 PBMC (male and female) colored by condition-specific signature scores.

Additional file 1: Figure S9. Sampling time scRNA-seq signature correction. (a) tSNE embedding of CLL cells from two donors before (left) and after (right) regressing out the time score. **(b)** Assessment of the computational correction robustness by bootstrapping datasets with varying compositions of affected cells. Silhouette width is inversely proportional to the intermixing of the conditions.

Additional file 1: Figure S10. Culturing PBMC removed the sampling time-associated bias.
(a, b) tSNEs showing the effect of culturing and CD3-activation of PBMC over two days on donors 2

(a) and 3 (b). **(c)** Time score distribution across sampling times with (orange) or without (blue) culturing and activating PBMC prior to processing.

Additional file 1: Figure S11. The impact of culturing and CD3-activating PBMC on their scRNA-seq profiles. (a,b,c) tSNEs embeddings displaying wheter or not cells were cultured (a), cell type annotation (b) and cell cycle score (c, S phase) of PBMC coming from 3 different donors (0h). **(d)** Cell type composition analysis in culutured and uncultured PBMC across 3 donors.

Additional file 1: Figure S12. The impact of sampling time at 4ºC on scRNA-seq profiles. (**a**) tSNE embedding of 265 CD3+ cells cryopreserved (i) immediately after blood extraction, (ii) storage at 4ºC or (iii) at 21ºC for 24/48 h prior to cryopreservation (Smart-seq2). Cells are colored by cluster label (SC3). (**b**) Distribution of processing conditions across cell clusters. **(c)** Volcano plot displaying the differentially expressed genes (DEG) in PBMC stored at 4ºC for 24h or 48h. **(d)** Number of DEG across sampling times for both 4ºC and 21ºC storage.

Additional file 1: Figure S13. (a,c,e) tSNE embedding of (**a**) PBMC, (**c**) CLL and (**e**) cultured PBMC colored by cell annotation. (**b**,**d**,**f**) tSNEs showing the selected markers for each of the cell annotations.

Additional Tables

Additional file 2: Table 1. Differentially expressed genes in prolonged (>2 h) storage of PBMC, CLL, T-cells, NK, monocytes and B-cells.

Additional file 3: Table 2. Enriched Gene Ontology (GO) terms in storage time-dependent DEG in PBMC, CLL, T-cells, NK, monocytes and B-cells.

Additional file 4: Table 3. Gene signature associated to storage time in PBMC (measured by scRNA-seq and microarray), CLL (measured by scRNA-seq), and collagenase-dependent tissue dissociation (van den Brink).

Additional file 5: Table 4. Transcription factor binding site motif enrichment analysis at sampling time-sensitive enhancers.

Additional file 6: Table 5. Differentially expressed genes in cultured CD4+ T-cells, cytotoxic cells and B-cells activated with anti-CD3 antibody.

Additional file 7: Table 6. scRNA-seq quality control metrics stratified by time, donor and temperature in both experiments (PBMC and CLL). Number of cells for each cell type stratified by time, donor and temperature in both experiments (PBMC and CLL). scATAC-seq quality control metrics stratified by time, donor and temperature in both experiments (PBMC and CLL).

Supplementary Figure 1

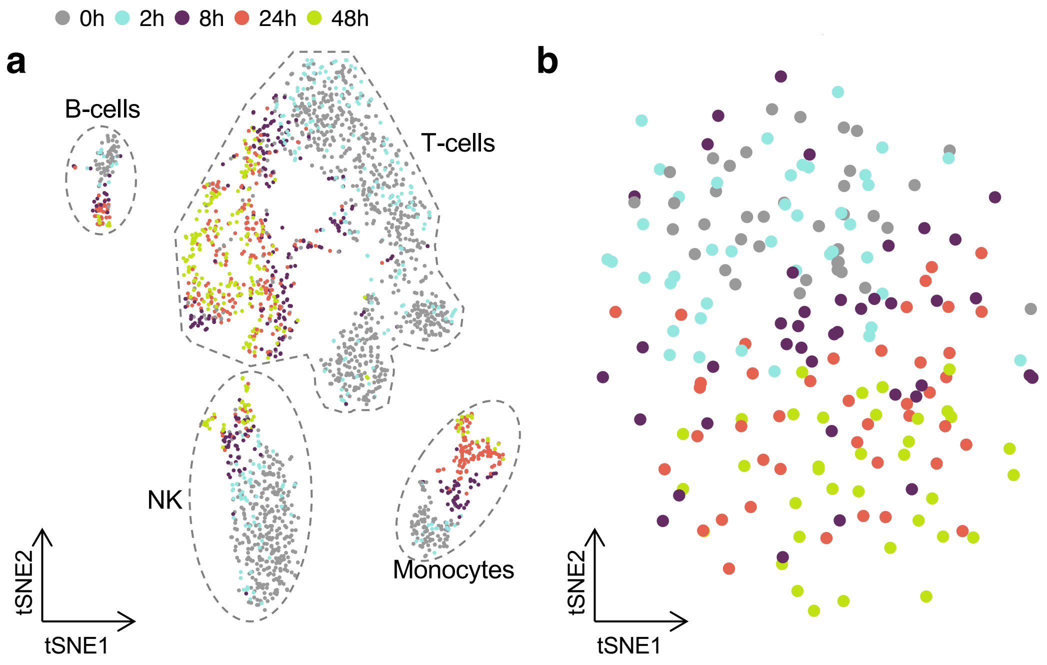

Supplementary Figure 2

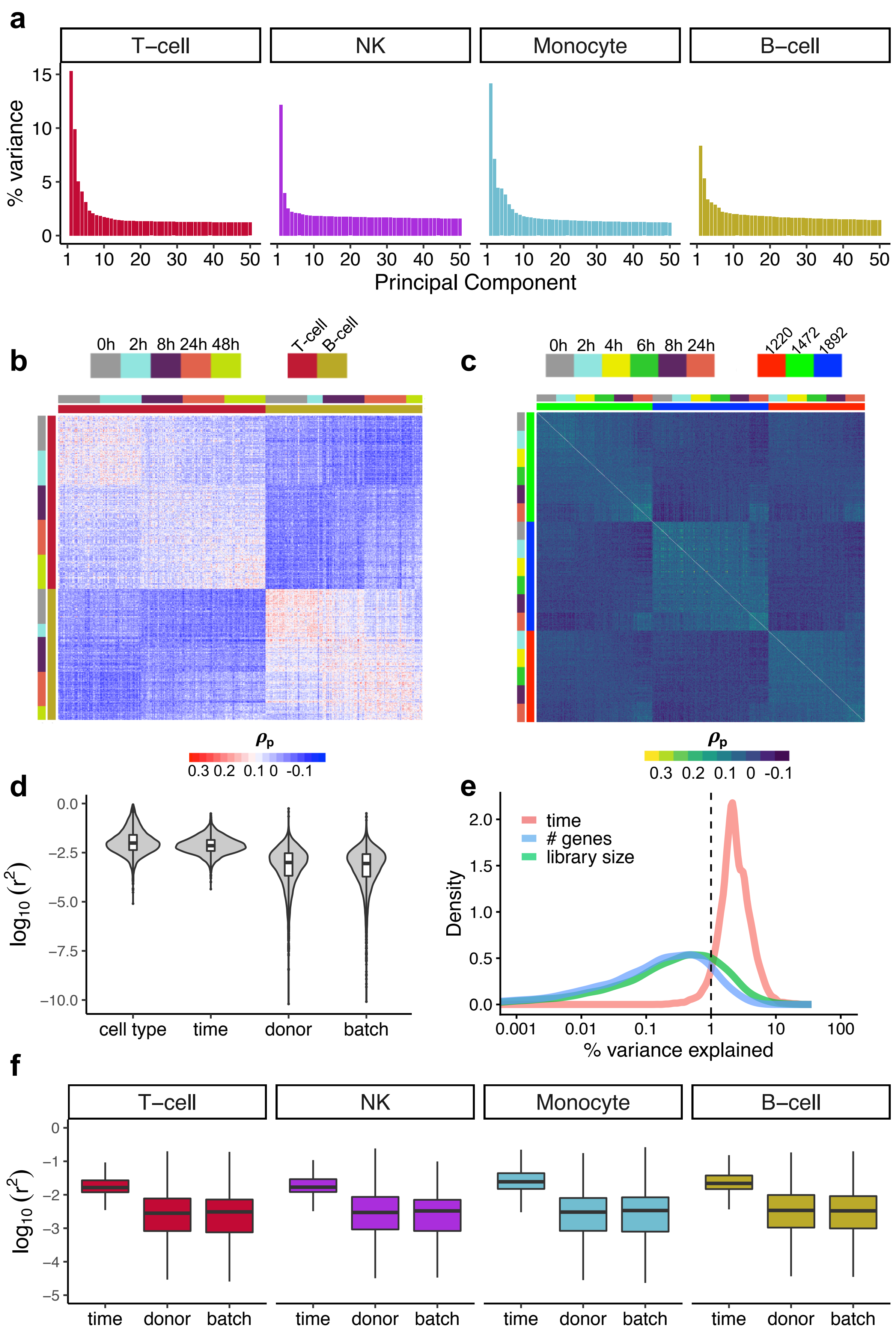

Supplementary Figure 3

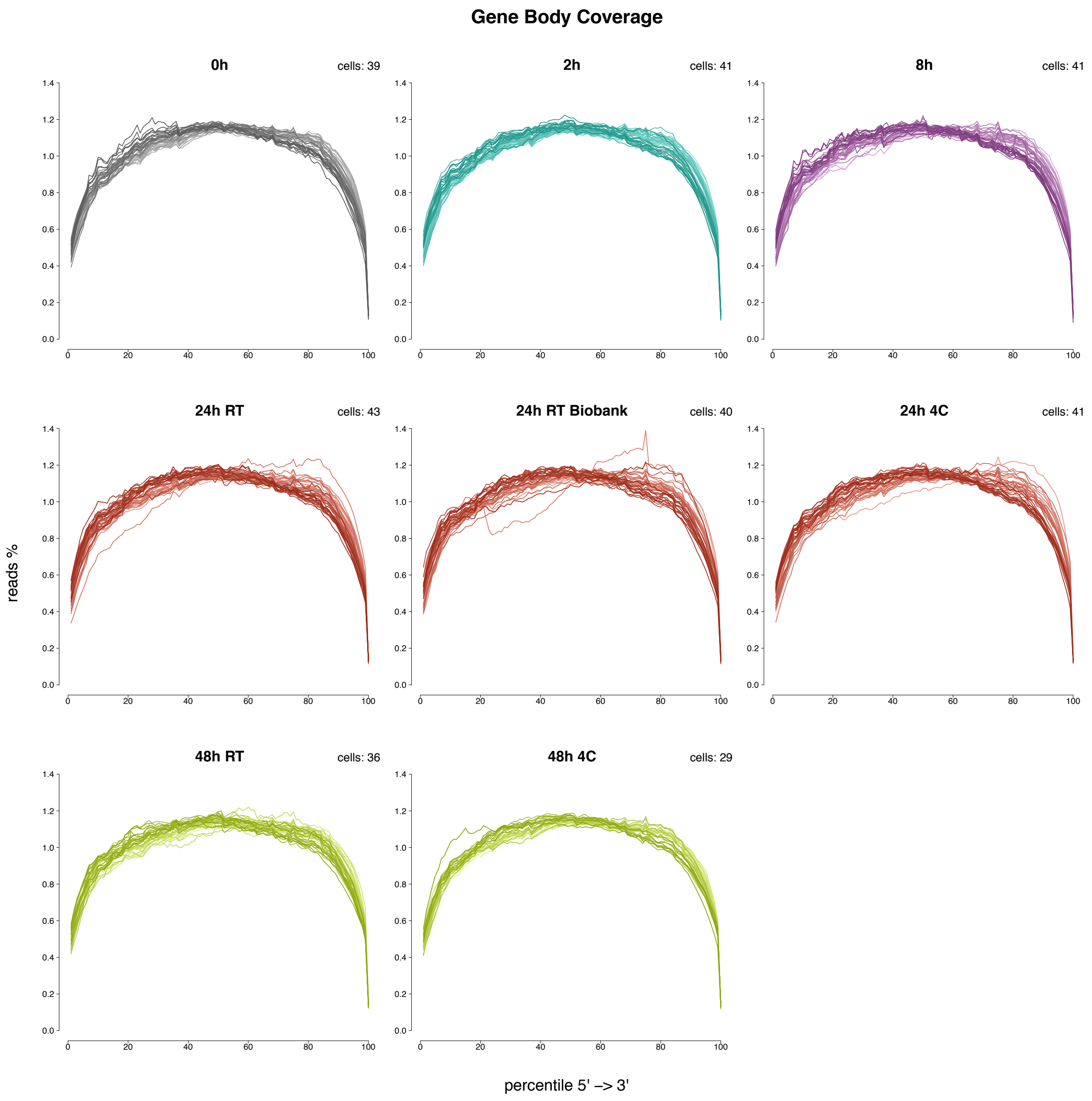

Supplementary Figure 4

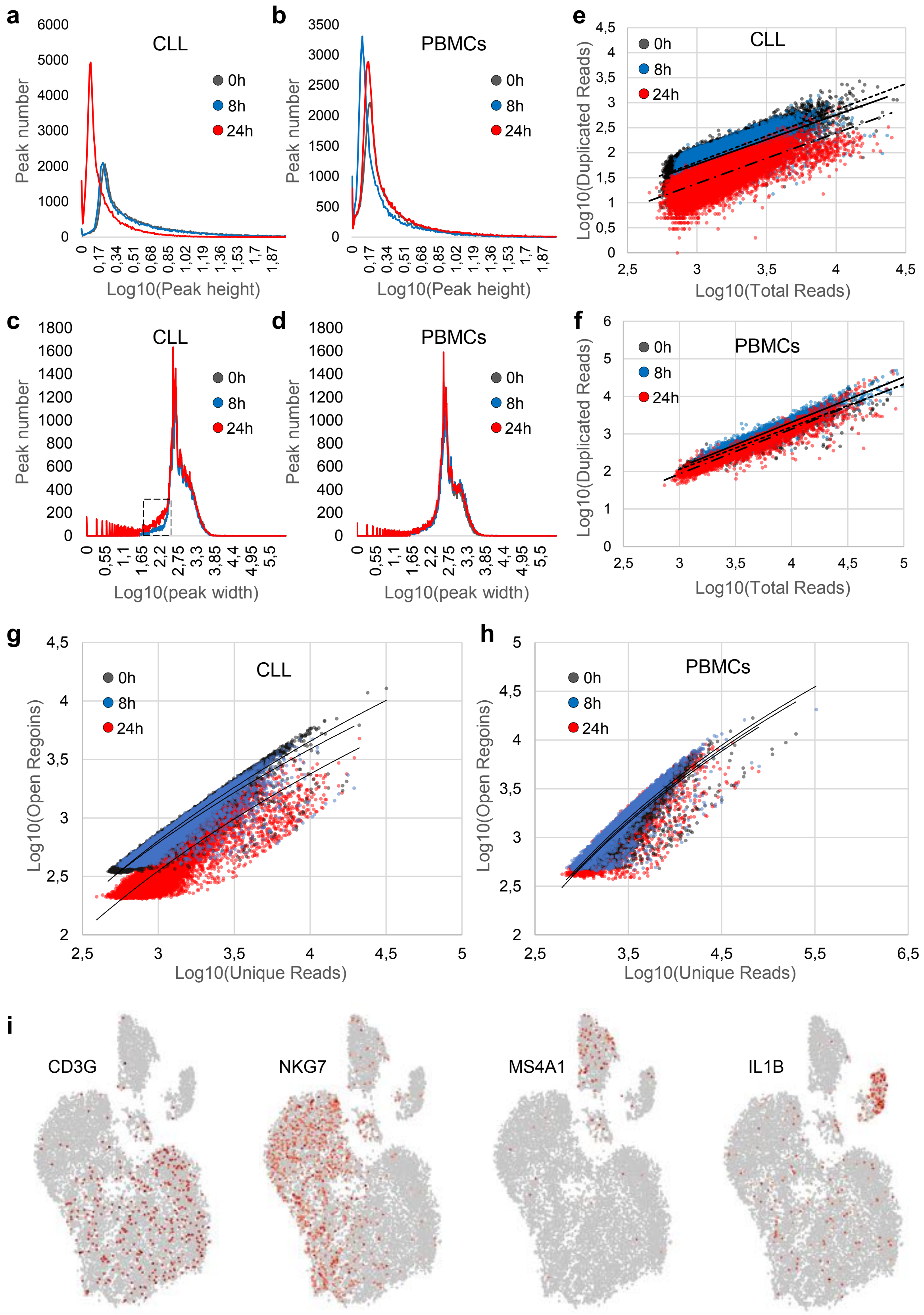

Supplementary Figure 5

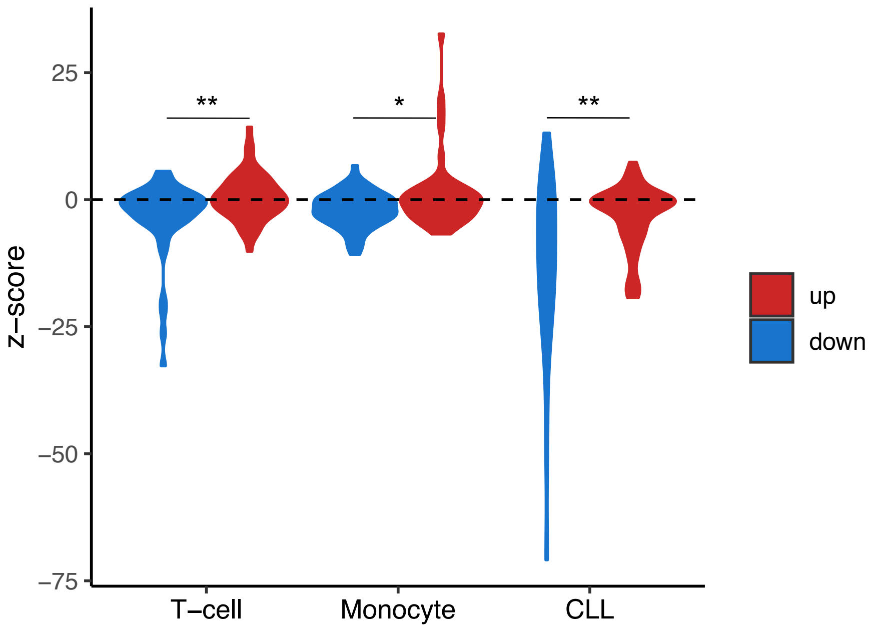

Supplementary Figure 6

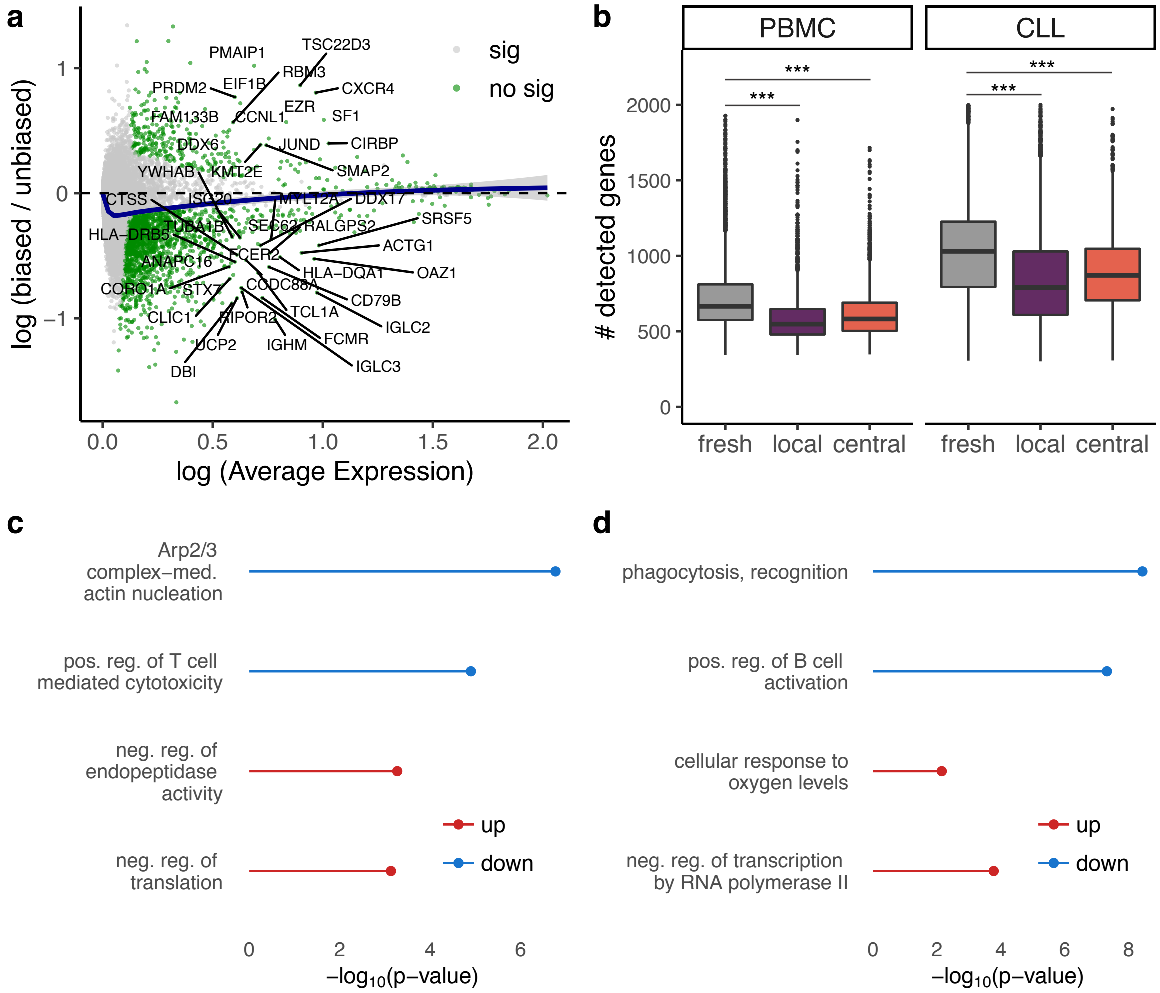

Supplementary Figure 7

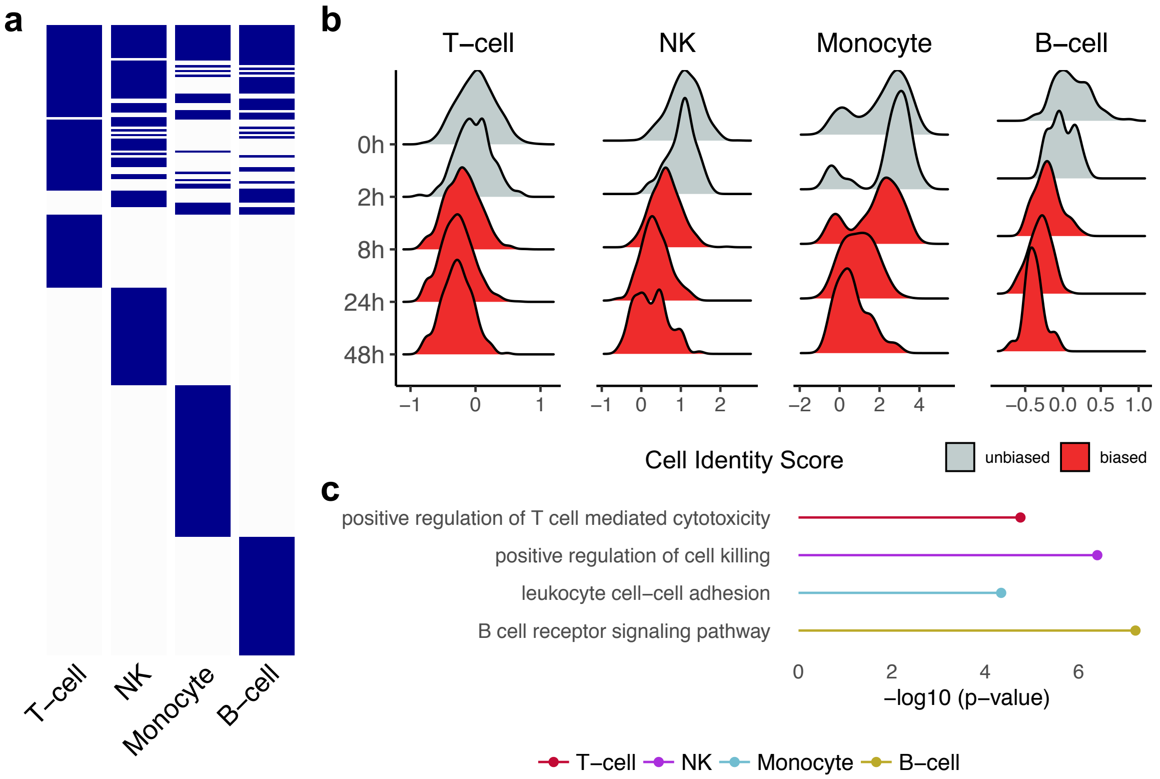

Supplementary Figure 8

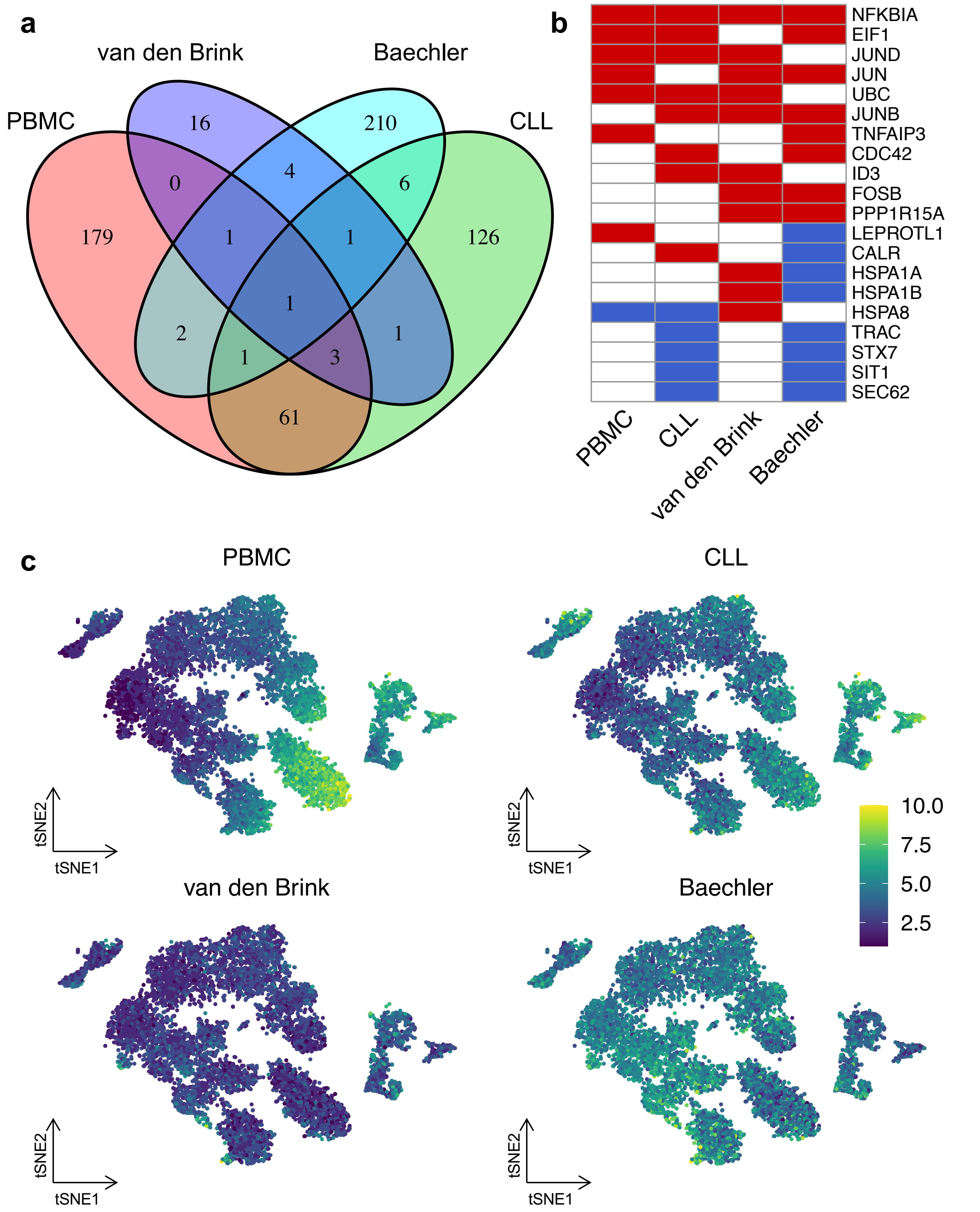

Supplementary Figure 9

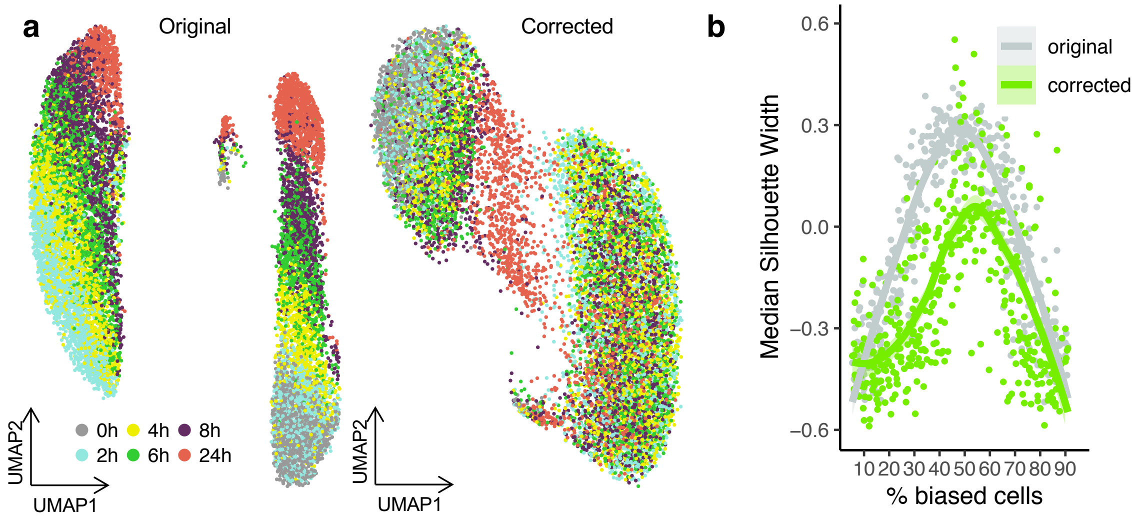

Supplementary Figure 10

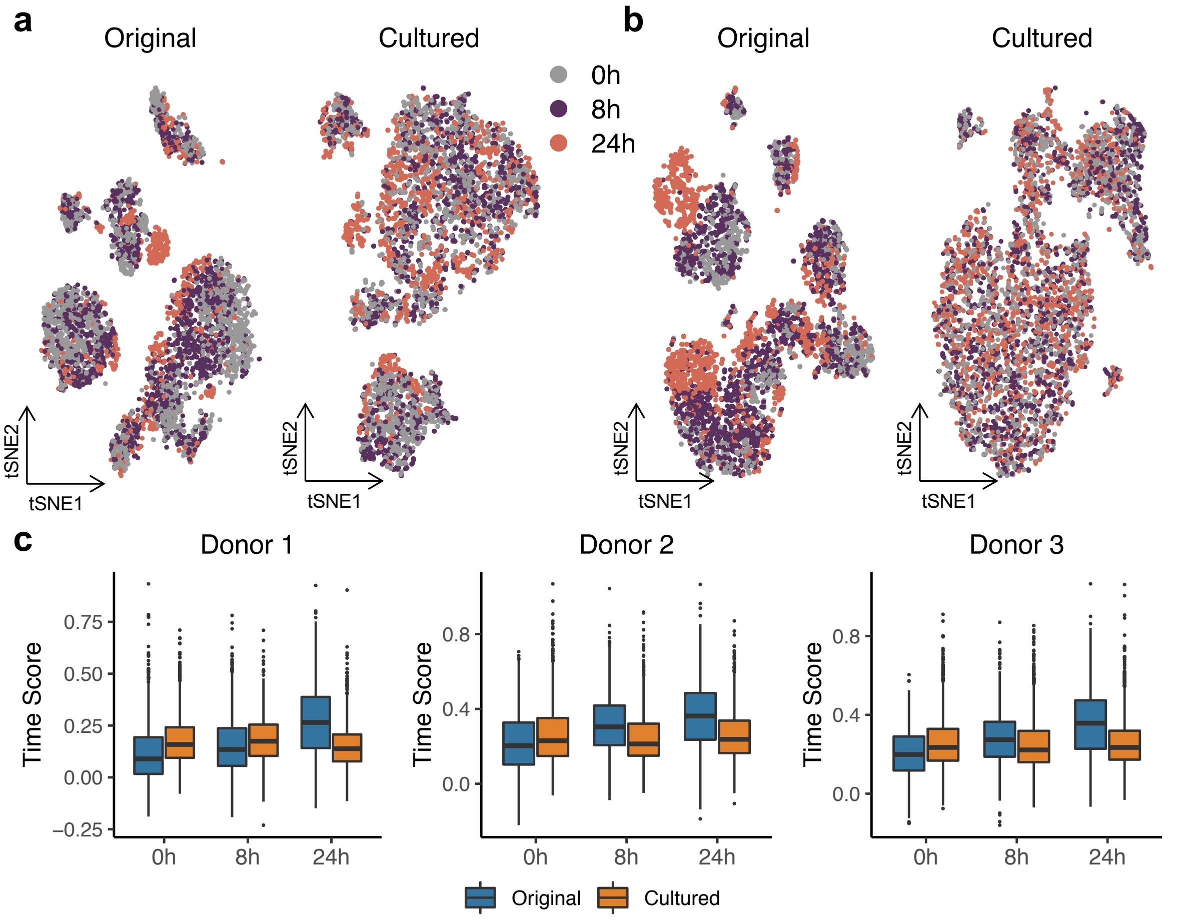

Supplementary Figure 11

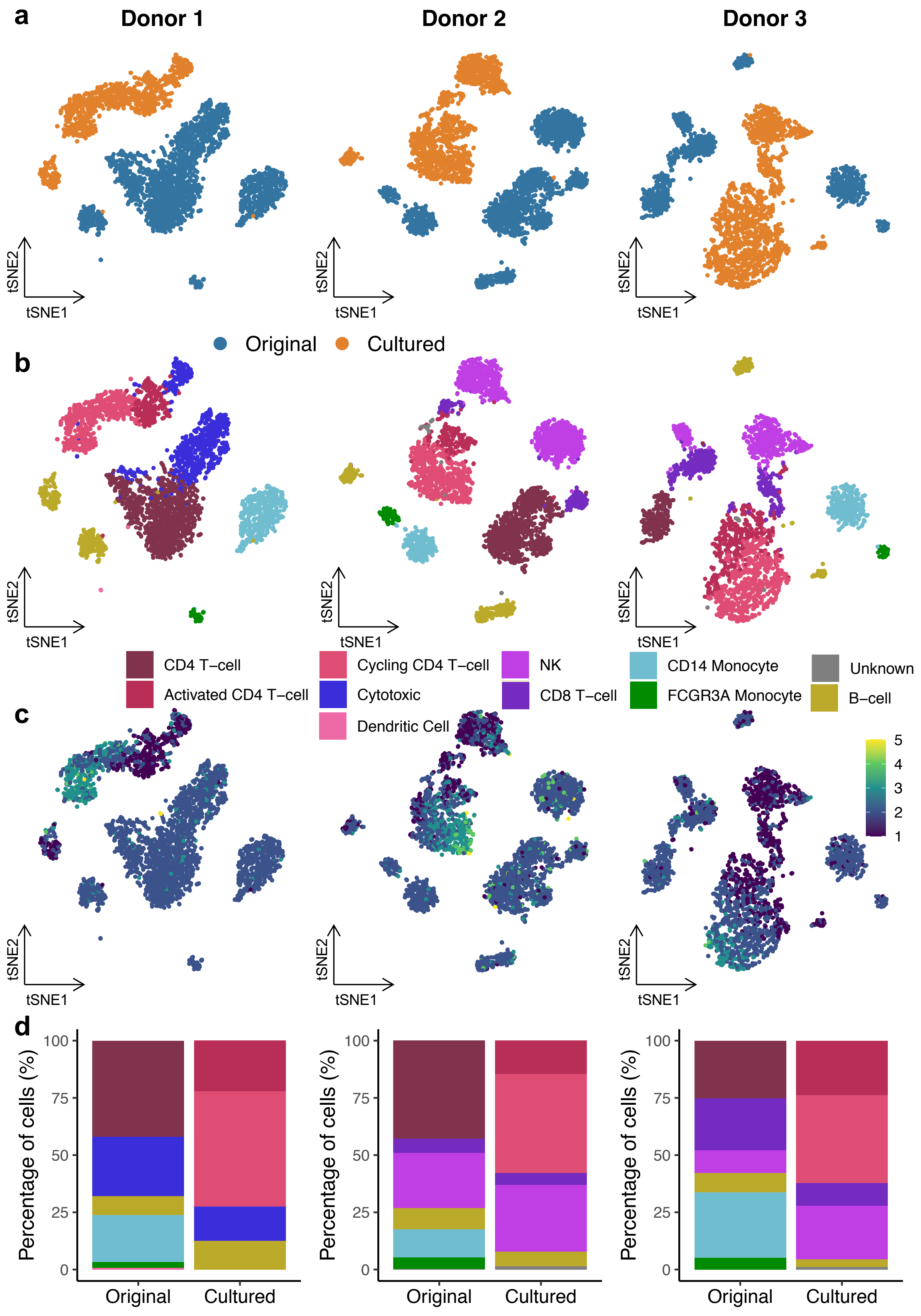

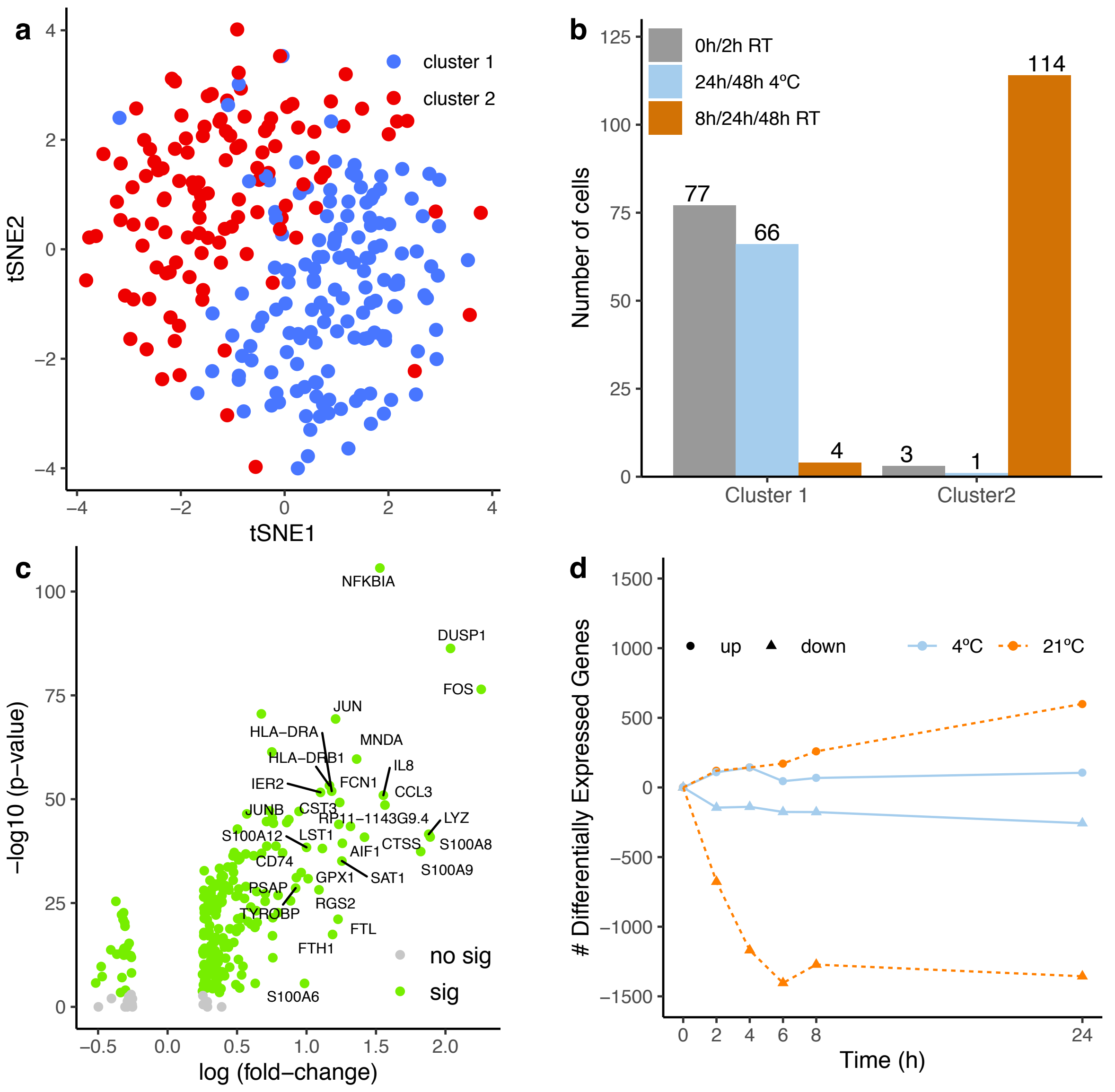
a
cluster 1
cluster 2
tSNE1
tSNE2
b
0h/2h RT
24h/48h 4ºC
8h/24h/48h RT
Number of cells
77
66
4
3
1
114
Cluster 1
Cluster2
c
−log10 (p−value)
log (fold−change)
NFKBIA
DUSP1
FOS
JUN
HLA−DRA
MNDA
HLA−DRB1
IL8
IER2
FCN1
CCL3
JUNB
CST3
RP11−1143G9.4
LYZ
S100A12
LST1
AIF1
CTSS
S100A8
CD74
S100A9
GPX1
SAT1
PSAP
RGS2
TYROBP
FTL
FTH1
S100A6
no sig
sig
d
up
down
4ºC
21ºC
Differentially Expressed Genes
Time (h)

Supplementary Figure 13

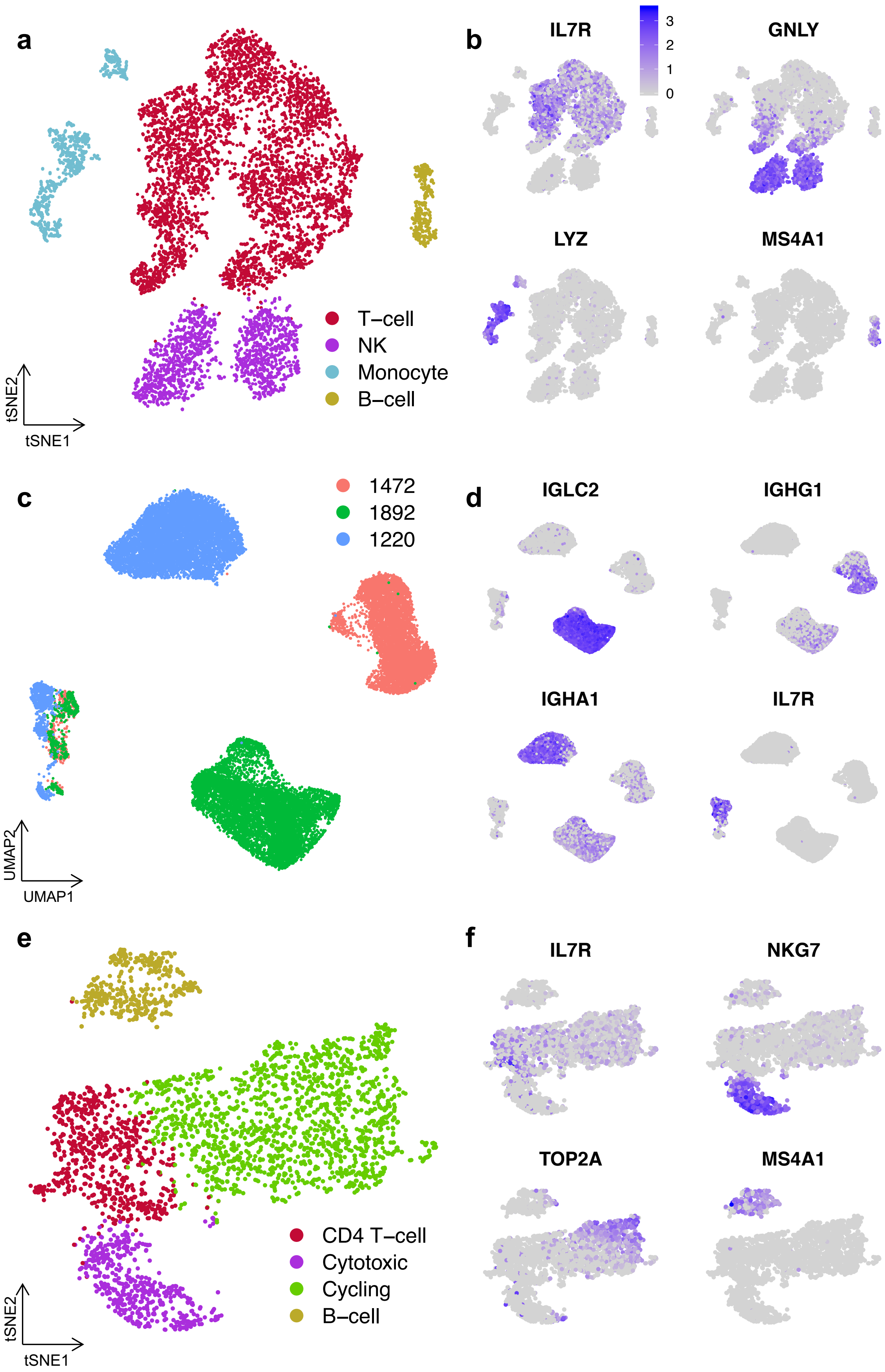

Supplement: Supplementary file 1 — Additional file 1: Supplementary material. Supplementary Figs. 1–13 and Supplementary figure and table legends. [file 13059_2020_2032_MOESM1_ESM.pdf]
