# Supplementary material for: Sampling time-dependent artifacts in single-cell genomics studies
Source: Genome Biol. 2020 May 11;21:112. doi: 10.1186/s13059-020-02032-0 (PMC7212672; doi:10.1186/s13059-020-02032-0)
Supplement: Supplementary file 8 — Additional file 8. Review history. [file 13059_2020_2032_MOESM8_ESM.docx]

Review History

**First round of review**

**Reviewer 1**

**Are you able to assess all statistics in the manuscript, including the appropriateness of statistical tests used?**

There are no statistics in the manuscript.

**Comments to author:**

This study examines the scRNA-seq and scATAC-seq measured effects of storing blood at room temperature for periods from hours to days. The changes in gene expression are quite significant, even after only a few hours. Hundreds of genes showed significant changes in expression. Many of these genes reflected a "cold shock" response. Changes measured by scATAC-seq were more subtle.

It is actually, however, not all surprising that cells that have been allowed to sit at room temperature for hours to days would show a "cold shock" gene expression response. This would appear to be the main conclusion of the paper and based on the many historic studies of the room temperature "cold shock" response it would seem the entirely expected result. It also not surprising that if the stored cells are them grown in culture for two days there would be an erasure of the cold shock response. Two days in culture is a lot of time to re-normalize. And finally, it is shown that if samples are stored at cold temperatures (4 deg) then there were not detectable changes in gene expression, again as might be expected based on previous studies showing that even entire organs can be preserved on ice for up to 72 hours without detectable change in scRNA results (their ref 15).

My main criticism of this work, therefore, is that there is not really much new. Again, store at room temperature and you get a cold shock response, culture for two days and you remove it, and if instead you store cells at 4 deg then gene expression patterns are preserved. Nothing very novel here.

There is a listing of genes that specifically change in PBMCs as a function of room temperature storage, and an algorithm to provide some correction that could be of use for those dealing with these sorts of samples.

Other comments:

Two different 10X technologies were used, V2 and V3. There can give very different results, with V3 for example detecting many more genes called expressed per cell. Which samples were processed with which technology? Could this contribute to the observed differences? The manuscript needs to be more clear on this important point.

Results and Discussion. First paragraph line 51. It is stated that viable cells did not show signs of reduced integrity across the time points. But what percent cells were actually viable at the different time points? Related, it is stated that there are 66,136 and 76,146 high quality cells for scRNA-seq and scATAT-Seq, respectively. But how many for each time condition? It is stated in the Methods section that "… an equal number of cells was taken for each time condition." But the later times point, like 48 hours, seem to have very sparse data. Is this because there were few high quality/viable cells even though similar numbers were loaded onto the 10X? So how many "high" quality cells were actually recovered for each condition?? It is stated that "viable cells" did not show signs of reduced RNA integrity across the time points. Again, not a surprise, as would not really expect the RNA to degrade until after cell death, especially as measured by the standard RIN scores that monitor rRNA integrity.

Not a comment on this paper, but a general comment on the commonly used "cold shock" terminology. As used, "cold shock" refers to room temperature and not ice cold, which is confusing. Many studies, including this one, show that expression patterns are generally quite well preserved on ice, but not at room temperature, or in a foreign environment at 37 degrees.

**Reviewer 2**

**Are you able to assess all statistics in the manuscript, including the appropriateness of statistical tests used?**

Yes, and I have assessed the statistics in my report.

**Comments to author:**

In this manuscript by Massoni-Badosa et al., the authors describe how storing peripheral immune cells at room temperature for different periods of time impacts their expression profile and chromatin accessibility, using single-cell RNA-Seq (scRNA-Seq), and single-cell ATAC-Seq (scATAC-Seq), respectively. Blood samples collected from two healthy donors and three chronic lymphocytic leukemia (CLL) patients were stored at room temperature for 0 - 48 hours before processing. The authors analyze changes in single-cell expression or chromatin accessibility profiles across time, and report a significant effect of storage time ("sampling time") on single-cell expression profiles; a global effect of sampling time on chromatin accessibility profiles could not be detected. The authors describe the change in expression signature associated with this sampling time as possessing "characteristics of a cold-shock response". Finally, the authors apply different strategies to computationally remove the effect. It is worth noting that efforts to apply scRNA-Seq to virtually all tissues from humans and many other organisms are well under way, and a better understanding of the artifacts introduced by different sample preparation protocols is of key importance for these efforts.

Main comments:

1. The study presented in this manuscript focusses almost exclusively on a single protocol parameter, storage time at room temperature in a single tissue (PBMCs) from healthy donors and CLL patients. There is a lack of context; cursory references are made to two UK Biobank studies, which do not include any transcriptomic experiments in their design, and a 2017 study from their group that pertains to the cryopreservation of samples for scRNA-Seq. To address the importance of the issues investigated in the manuscript, the authors could discuss ongoing or planned efforts pertaining to large-scale analyses of blood samples with scRNA-Seq or scATAC-Seq that would benefit from lessons learned from this study. There is also insufficient discussion of background related to recent and ongoing investigations on effects of different sample preparation protocols on scRNA-Seq data (the Human Cell Atlas community is putting in a lot of effort toward this). It is important to reference a few additional and more recent studies, if only to provide a sense of the intense interest in this area of research. These studies could also be relevant to the interpretation of the results presented, and highlight the broad applicability of the work.

2. The manuscript title appears overly broad and fails to convey the main parameter tested, i.e., sampling time. Suggest editing the title to describe the actual parameter(s) tested and include some aspect of "time" (handling time? storage time?). "Sampling artifacts" is too broad and include other aspects of study design, e.g., preservation method, frequency of sampling (in a time-course experiment), choice of single-cell RNA-Seq/ATAC-Seq protocol, single-cell vs. single nucleus, etc. that are not tested here.

3. An important claim in the manuscript is that the observed time-dependent effect on the transcriptome is large, as it is being described as "striking" (p. 2, l. 42) and varying "profoundly" (p. 2, l. 52) which does not appear to be true. In the t-SNE visualizations shown, the effect seems to be dominated by differences between cell types (Fig. 1a) and donor (Fig. 1b). The authors perform regression analysis to show that storage time differences are associated with somewhere between 1-10% of variance for most genes (Fig S1c), however this analysis can be confounded by differences in cell type proportions between the samples, for example. The authors also show that after clustering cells by cell-type, there is a correlation between PC1 scores and storage time (Fig 1c), but the total proportion of variance explained is not quantified. Finally, the authors determine that between 200-236 genes showed statistical significance differential expression, but the associated effect sizes (fold changes) appear quite small, well below 2-fold (Fig 1g). The authors could try to clarify the magnitude of the observed effect in a number of ways, including:

a. Introduce a universal measure of cell similarity, e.g., Pearson correlation of expression profiles after log-transform, and use this measure to quantify the difference between cells from different conditions. This would allow a quantitative comparison of the differences e.g., between T cells before and after storage for 24h, with the differences between cell types, e.g., between T cells and B cells. Since Pearson correlation is a universal measure, it would be easy to compare the results obtained from different experiments (e.g., CLL and healthy PBMCs).

b. Perform a regression analysis (as in Fig S1c), but separately for each cell type, in order to avoid confounding with differences in cell type proportions (alternatively, ensure that the cell type proportions are identical across all datasets). For each cell type, add up the amount of variance explained by storage time for each gene, and compare this to the total amount of variance observed, both among the cells from each cell type, as well as globally (variance observed when including all cells) to obtain a measure of the proportion of cell type-specific variance explained by the effect, as well as the proportion of total variance explained.

c. Directly compare the magnitude of the observed effect with other condition-specific effects, perhaps using data from other studies that measure different artifacts (e.g., the effect of cold preservation examined in the 2017 study from the same group).

4. The authors attempt to characterize the observed effect as a "cold-shock response" by computational means, but the analyses are not very convincing and no experimental validation is performed. The authors write that they observe a "global downregulation of gene expression" (p3 l. 19) but it is not clear from the results presented if this is accurate, with only <200 downregulated genes, and a slight apparent reduction in average expression levels (please describe what the blue lines in Figure 1g and S6a indicate). The authors mention of a similar effect being observed in a 2004 bulk transcriptomic study by Baechler et al. (ref. 5), however no direct comparison with the data from this study is performed. It is also unclear how the observed expression signature relates to previously reported "dissociation artifact" in scRNA-Seq data (Brink et al., ref. 9), which the authors cite later in a different context. Since the Brink et al. study involves scRNA-Seq data, and there appear to be some parallels in terms of genes induced (such as FOS and JUN), it would be important to perform a more detailed comparison with data from that study.

There are two issues with the gene ontology enrichment analysis: 1) the GO terms reported as enriched appear only indirectly related to the cold-shock response, 2) the authors show enrichment p-values of four GO terms for the PBMC and CLL datasets, respectively (Figure S6c,d). Are those GO terms the only ones with significant p-values? If not, it would be helpful to provide the full lists of significantly enriched GO terms, as a context for interpreting the results.

5. In the last part of the manuscript, the authors discuss strategies for addressing or avoiding the observed artifact which can be potentially very useful to the single cell community. While the authors propose a range of strategies, each one is studied and discussed in relatively little detail, and it ultimately unclear what the conclusions are of this part of the study and what bearing they may have for future study design. The authors correctly point out that regression-based approaches can be problematic, as they may accidentally remove signals of biological interest (p4, ll. 10-13). The authors also show that the artifact in PBMCs was greatly reduced after culturing for two days (Fig 2c,d), however they do not study how the culturing of primary immune cells affects their cell type composition and cell type-specific expression patterns. It is therefore unclear in what situations this approach would be useful. Finally, the authors follow up on a recent report that solid tissues can be effectively preserved at 4 degrees Celsius ("cold storage", Madissoon et al., ref. 15), and test for the presence of the artifact after cold storage. They show that this preservation appears to eliminate the expression artifact (Figure 2e,f). Out of all the strategies studied, this approach would appear to hold the most promise for practical applications, but unfortunately the authors only present only a cursory analysis of the data. For example, it would be important to analyze the expression profile of the genes that form the previously identified expression signature of the storage artifact. Furthermore, it is not clear how these experiments were designed, as the manuscript refers to cells "stored at 4°C (24h/48h)". Which cells were stored for 24h and which for 48h, and whether they were combined for analysis purposes only, or immediately after cell isolation, does not seem to be indicated anywhere.

Minor comments:

1. Basic metrics relating to the experiments are missing, in particular, the number of cells obtained for each time point (before and after QC), and the number of cells belonging to each cell type. Maybe these could be provided in a supplementary table.

2. How do these numbers/ fractions of cell-types in PBMC compare to previous studies using single cell genomics, flow cytometry, etc.? Do the authors have any comments on inter-individual variability in terms of number fractions of each cell-type in PBMC? Or are such observations confounded by technical challenges and cannot be made with confidence?

2. Comments for Figure 1:

a. How many cells in each condition for Figures a,b,d?

b. While the visualizations in panels a/b/d broadly convey the main results described in the paper, some aspects are problematic:

i. In panels a and b, cell diameters are very large so that most cells are probably completely occluded from the reader's view. This could be improved by redrawing the plots with a smaller cell diameter, as in panel d.

ii. Also, it appears as if cells from certain time points are occluded behind cells from other time points (e.g., very few cells from the 2h time point are visible in a and likely occluded behind cells from the 0h time point). If this is the case, this could be improved by randomizing the order in which cells are drawn (depending on the visualization tool used, this may be technically challenging - in this case reducing the cell diameter should at least alleviate the problem; see above).

iii. Since color is used to indicate the time point, the cell type assignments are not clear. This could be improved by manually drawing boundaries around each cluster. If clusters do not cleanly separate by location in the t-SNE plot, an approximate boundary could be indicated with a dashed line to improve the readability of the figure.

c. It is unclear whether panel c shows results from a single (global) PCA, for cells grouped by cell type, or whether a PCA was conducted for each cell type separately. This could be clarified in the figure legend.

d. For panel e, it is unclear what the "p-value in z-score scale" means. If the values represent z-scores, the label of the y-axis should simply read "z-score".

e. In panel e, significance thresholds based on p-values are displayed, but it is not clear what statistical test was used to obtain those p-values. This information should be included in the figure legend.

f. In reference to panel e and Figure S4, the claim is made that "Specifically, we detected reduced expression for genes that lose open chromatin sites, a trend that was more pronounced analyzing enhancers compared to promoter sites [...] and suggesting that specific enhancer sets trigger the response to temperature changes." (p3 ll. 1-4) However the authors do not quantify this effect or test its significance, and apparently the claim is based simply on a visual comparison between the violin plots, which is quite subjective. It would therefore seem appropriate to qualify this claim, provide additional evidence in the form of quantitative analyses, or omit this claim from the manuscript as it does not appear to be a central result.

In summary, the study focuses on an important question related to blood sample preparation for scRNA-Seq analysis, and the experimental and computational methodologies applied appear technically sound. However, it is unclear exactly what the implications of the presented results are for the design of future scRNA-Seq studies. A discussion on the generalizability of the findings and methods suggested to bypass the technical challenges to other systems will be helpful.

**Authors Response**

**Point-by-point responses to the reviewers’ comments:**

Response to Reviewers:

Reviewer #1: This study examines the scRNA-seq and scATAC-seq measured effects of storing blood at room temperature for periods from hours to days. The changes in gene expression are quite significant, even after only a few hours. Hundreds of genes showed significant changes in expression. Many of these genes reflected a "cold shock" response. Changes measured by scATAC-seq were more subtle.

It is actually, however, not all surprising that cells that have been allowed to sit at room temperature for hours to days would show a "cold shock" gene expression response. This would appear to be the main conclusion of the paper and based on the many historic studies of the room temperature "cold shock" response it would seem the entirely expected result. It also not surprising that if the stored cells are them grown in culture for two days there would be an erasure of the cold shock response. Two days in culture is a lot of time to re-normalize. And finally, it is shown that if samples are stored at cold temperatures (4 deg) then there were not detectable changes in gene expression, again as might be expected based on previous studies showing that even entire organs can be preserved on ice for up to 72 hours without detectable change in scRNA results (their ref 15).

My main criticism of this work, therefore, is that there is not really much new. Again, store at room temperature and you get a cold shock response, culture for two days and you remove it, and if instead you store cells at 4 deg then gene expression patterns are preserved. Nothing very novel here.

There is a listing of genes that specifically change in PBMCs as a function of room temperature storage, and an algorithm to provide some correction that could be of use for those dealing with these sorts of samples.

*Response: We agree and disagree with the Reviewer. Indeed, this is not the first study describing the cold-shock response in blood cells through transcriptome analysis. Previous work in the field has identified such effect using microarray or bulk RNA-seq analysis. It is important to note though that previous studies did not have the resolution provided in our work, where we use single-cell RNA-seq and ATAC-seq in a thoroughly designed benchmarking study a) to assess time-dependent biases at several consistent sampling intervals across replicates in healthy and diseased samples, b) to provide mechanistic insight integrating transcriptome and epigenome data, c) to establish a scoring system to identify affected samples, d) to experimentally and computationally solve the problem for prospective and retrospective study designs. Most importantly, we provide a reference work to the wealth of ongoing and future blood-based single-cell cohort studies. We expect large single-cell cohort studies, some now mentioned in the Introduction of the manuscript, to benefit from this work by adjusting their sampling strategies to avoid major artifact in the data and false conclusions. Single-cell genomics is a powerful approach to revisit existing clinical cohorts and to ask novel questions about inter-individual variability (population cohorts). We foresee an explosion of PBMC-based datasets in this area. Our work should provide the basis for a high-quality data production to maximize outcomes.*

Other comments:

1. Two different 10X technologies were used, V2 and V3. There can give very different results, with V3 for example detecting many more genes called expressed per cell. Which samples were processed with which technology? Could this contribute to the observed differences? The manuscript needs to be more clear on this important point.

*Response: We apologize for the lack of clarity on the usage of different 10x Genomics scRNA-seq versions in this study. The revised manuscript contains a detailed description which version was used to generate datasets. It is very important to state that we never compared results across experiments conducted with different versions. In contrast, we generated all datasets across time point without any batch effect through the use of cell hashing and sample multiplexing. All sample had been cryopreserved and could be processed in a single experiment. Therefore, we labeled samples from different sampling time points with indexed antibodies (cell hashing) and multiplexed all conditions into a single 10X Genomics run. Consequently, all condition entered the same amplification pool, avoiding batch effects during library preparation. After sequencing, cells were assigned to their respective condition with their sample indexes. We have modified the main text and Methods to state this design more clearly.*

*“In this work, we designed benchmarking experiments to systematically test the effect of varying processing times on single-cell transcriptome and epigenome profiles from healthy and diseased donors, while controlling for technical variability (e.g. batch effects; Methods).”*

*“Sequencing libraries were prepared using the single-cell 3’ mRNA kit (V2 for PBMC samples and V3 for CLL and cultured PBMC samples; 10x Genomics) with some adaptations for cell hashing, as indicated in TotalSeq™-A Antibodies and Cell Hashing with 10x Single Cell 3' Reagent Kit v3 3.1 Protocol by BioLegend.”*

2. Results and Discussion. First paragraph line 51. It is stated that viable cells did not show signs of reduced integrity across the time points. But what percent cells were actually viable at the different time points? Related, it is stated that there are 66,136 and 76,146 high quality cells for scRNA-seq and scATAT-Seq, respectively. But how many for each time condition? It is stated in the Methods section that "… an equal number of cells was taken for each time condition." But the later times point, like 48 hours, seem to have very sparse data. Is this because there were few high quality/viable cells even though similar numbers were loaded onto the 10X? So how many "high" quality cells were actually recovered for each condition?? It is stated that "viable cells" did not show signs of reduced RNA integrity across the time points. Again, not a surprise, as would not really expect the RNA to degrade until after cell death, especially as measured by the standard RIN scores that monitor rRNA integrity.

*Response: We did not observe a decrease in viability at later time points after defreezing of the cryopreserved samples. Indeed, all time points showed very high viability (average 95%, range: 88-98%). The viability has been quantified by viability staining (DAPI) in the FACS and is now included as additional text in the Methods. However, we noticed the total cell fraction to decrease over time due to a possible cell lysis during prolonged storage time. A decreased cell fraction could have influenced the quantification before sample multiplexing, resulting in unbalanced sample pools. Inspecting the total number of cell per time point, we could not detect a systematic decrease in the later time points. Although, especially the PBMC samples have not been perfectly balanced, a clear bias could be detected against the samples stored for 24 or 48 hours. The total number of cells per sample and time point are now included in a new Supplementary Table 6. The table also includes the number of cells removed during data quality control, which was generally very low. Here, we could not detect an increased frequency of damaged/dead cells in the later time points.*

*“Of note, we did not observe an increase of damaged/dead cells (cell viability staining) towards the later time points with an average viability of 95% (range: 88-98%) across time points. An increased fraction of debris could be observed at 24 and 48 hours, which, however, did not result in reduced data quality (e.g. proportion of excluded cells) during data analysis (Additional file 7: Table 6).”*

*“Of note, all sampling time point showed comparable dataset qualities (e.g. library size and mitochondrial read content; Additional file 7: Table S6).”*

*“We provide a supplementary table with the number of cells before and after QC, the average library size, library complexity and mitochondrial fraction stratified by time, donor and temperature (Additional file 7: Table 6).”*

*“We provide a supplementary table with the number of cells per cell type stratified by time, donor and temperature (Additional file 7: Table 6).”*

*“We provide a supplementary table with multiple QC metrics (number of high-quality cells and detected peaks, among others) stratified by experiment, donor and time (Additional file 7: Table 6).”*

3. Not a comment on this paper, but a general comment on the commonly used "cold shock" terminology. As used, "cold shock" refers to room temperature and not ice cold, which is confusing. Many studies, including this one, show that expression patterns are generally quite well preserved on ice, but not at room temperature, or in a foreign environment at 37 degrees.

*Response: We agree with the Reviewer that the term “cold shock” is misleading, especially when the study experimental design also includes cells stored on ice. Therefore, we annotated the “cold shock score” as “time score” throughout the revised manuscript when appropriate. We kept the term “cold shock response” though in the interpretation of the gene expression signature.*

Reviewer #2: In this manuscript by Massoni-Badosa et al., the authors describe how storing peripheral immune cells at room temperature for different periods of time impacts their expression profile and chromatin accessibility, using single-cell RNA-Seq (scRNA-Seq), and single-cell ATAC-Seq (scATAC-Seq), respectively. Blood samples collected from two healthy donors and three chronic lymphocytic leukemia (CLL) patients were stored at room temperature for 0 - 48 hours before processing. The authors analyze changes in single-cell expression or chromatin accessibility profiles across time, and report a significant effect of storage time ("sampling time") on single-cell expression profiles; a global effect of sampling time on chromatin accessibility profiles could not be detected. The authors describe the change in expression signature associated with this sampling time as possessing "characteristics of a cold-shock response". Finally, the authors apply different strategies to computationally remove the effect. It is worth noting that efforts to apply scRNA-Seq to virtually all tissues from humans and many other organisms are well under way, and a better understanding of the artifacts introduced by different sample preparation protocols is of key importance for these efforts.

Main comments:

1. The study presented in this manuscript focuses almost exclusively on a single protocol parameter, storage time at room temperature in a single tissue (PBMCs) from healthy donors and CLL patients. There is a lack of context; cursory references are made to two UK Biobank studies, which do not include any transcriptomic experiments in their design, and a 2017 study from their group that pertains to the cryopreservation of samples for scRNA-Seq. To address the importance of the issues investigated in the manuscript, the authors could discuss ongoing or planned efforts pertaining to large-scale analyses of blood samples with scRNA-Seq or scATAC-Seq that would benefit from lessons learned from this study. There is also insufficient discussion of background related to recent and ongoing investigations on effects of different sample preparation protocols on scRNA-Seq data (the Human Cell Atlas community is putting in a lot of effort toward this). It is important to reference a few additional and more recent studies, if only to provide a sense of the intense interest in this area of research. These studies could also be relevant to the interpretation of the results presented, and highlight the broad applicability of the work.

*Response: We fully agree with the Reviewer about the importance of sample preparation benchmarking in single-cell studies and the impact on large-scale international consortia. Accordingly, we have added more background on current initiatives and previous benchmarking efforts for single-cell genomics approaches in the Introduction of the revised manuscript. Although the length of this Genome Biology format is limited in text and references, we believe the extra information is important to include and hope for the permission of the editor.*

*“However, standardizing sampling conditions is of crucial importance when designing single-cell genomics experiments to avoid technical artifacts in datasets and the misinterpretation of the results. Especially, large-scale consortia with multi-center sampling strategies, such as the Human Cell Atlas project [4] or the single-cell eQTLGen consortium [5], require dedicated standardization efforts to allow an informed decision-making process for guidelines and standards towards high quality data production. Previous work to determine sampling artifacts in scRNA-seq datasets identified profound alterations of gene expression signatures during sample preparation, storage and processing, strongly underlining the importance of specific benchmarking efforts [6–9].”*

2. The manuscript title appears overly broad and fails to convey the main parameter tested, i.e., sampling time. Suggest editing the title to describe the actual parameter(s) tested and include some aspect of "time" (handling time? storage time?). "Sampling artifacts" is too broad and include other aspects of study design, e.g., preservation method, frequency of sampling (in a time-course experiment), choice of single-cell RNA-Seq/ATAC-Seq protocol, single-cell vs. single nucleus, etc. that are not tested here.

*Response: We have modified the title to:*

*“Sampling time-dependent artifacts in single-cell genomic studies”*

3. An important claim in the manuscript is that the observed time-dependent effect on the transcriptome is large, as it is being described as "striking" (p. 2, l. 42) and varying "profoundly" (p. 2, l. 52) which does not appear to be true. In the t-SNE visualizations shown, the effect seems to be dominated by differences between cell types (Fig. 1a) and donor (Fig. 1b). The authors perform regression analysis to show that storage time differences are associated with somewhere between 1-10% of variance for most genes (Fig S1c), however this analysis can be confounded by differences in cell type proportions between the samples, for example.

The authors also show that after clustering cells by cell-type, there is a correlation between PC1 scores and storage time (Fig 1c), but the total proportion of variance explained is not quantified. Finally, the authors determine that between 200-236 genes showed statistical significance differential expression, but the associated effect sizes (fold changes) appear quite small, well below 2-fold (Fig 1g).

The authors could try to clarify the magnitude of the observed effect in a number of ways, including:

a. Introduce a universal measure of cell similarity, e.g., Pearson correlation of expression profiles after log-transform, and use this measure to quantify the difference between cells from different conditions. This would allow a quantitative comparison of the differences e.g., between T cells before and after storage for 24h, with the differences between cell types, e.g., between T cells and B cells. Since Pearson correlation is a universal measure, it would be easy to compare the results obtained from different experiments (e.g., CLL and healthy PBMCs).

b. Perform a regression analysis (as in Fig S1c), but separately for each cell type, in order to avoid confounding with differences in cell type proportions (alternatively, ensure that the cell type proportions are identical across all datasets). For each cell type, add up the amount of variance explained by storage time for each gene, and compare this to the total amount of variance observed, both among the cells from each cell type, as well as globally (variance observed when including all cells) to obtain a measure of the proportion of cell type-specific variance explained by the effect, as well as the proportion of total variance explained.

c. Directly compare the magnitude of the observed effect with other condition-specific effects, perhaps using data from other studies that measure different artifacts (e.g., the effect of cold preservation examined in the 2017 study from the same group).

*Response: We agree with the Reviewer that the variance introduced by storage time needs to be quantified more thoroughly and compared with other drivers of variance. We also thank the Reviewer for the actionable and thoughtful ideas to achieve the aforementioned objectives. To this end, we performed the following analysis.*

*¬¬First, we quantified the percentage of the total variance explained by the first 50 principal components (PC), for each of the 50 PC in each cell type. This revealed that PC1 (which correlates with sampling time; Fig 1d) explained between 15.3 % (T-cells) and 8.4 % (B-cells) of the variance (Supplementary Fig. 2a) and being much larger than the rest of PC.*

*Second, and following the Reviewer’s suggestion, we introduced ρp as a universal measure of cell similarity. In a benchmark of 17 cell-cell association metrics (Skinnider et al.), measures of proportionality (ρp and ɸs) displayed the highest accuracy (considerably better than Pearson correlation). Thus, we downsampled T-cells and B-cells from the PBMC dataset and leukemic cells from the three patients in the CLL dataset (see Methods), and computed all cell-cell pairwise ρp, yielding a distance matrix. Consistently with the qualitative tSNE visualization, cell type was the largest source of variance followed by sampling time (Supplementary Fig. S2b). Likewise, the donor was the largest driver of variance in the CLL datasets, consistent with the large inter-patient variability observed in CLL (Supplementary Fig. S2c).*

*Finally, for the PBMC datasets, we regressed the gene expression values of 5,282 genes on one of four explanatory variables (cell type, time, donor and batch), and extracted a distribution of r2 values for each variable. The order of magnitude of r2 was very low due to the inherent sparsity of scRNA-seq data (<0.1% of variance for the vast majority of genes in all variables). However, the overall distributions allowed for a faithful ranking of drivers of variance, starting with cell type (3.41% on average), and followed by sampling time (1.25% on average), donor (0.28% on average) and batch (0.26% on average; Supplementary Fig. S2d). This was confirmed with a similar analysis on T-cells measured by Smart-seq2, where time was again a larger driver of variance than other technical variables (Supplementary Fig. S2e). Finally, down-sampling and stratifying by cell type revealed the same trend (Supplementary Fig. S2f).*

*In light of the above, we still consider sampling time to be a major potential confounder in scRNA-seq data that, if not considered, might lead to serious misinterpretations. Nevertheless, it is not the major source of variance in neither PBMC nor CLL datasets. Thus, we have moderated our language and removed the words “striking” and “profoundly” from the main text.*

*Modification in the main text:*

*“Sampling time correlated with the first principal component (PC1) for all cell subtypes (Fig. 1c), explaining between 15.3% (T-cells) and 8.4% (B-cells) of the variance contained in the first 50 PC (Additional file 1: Fig. S2a). Moreover, sampling time followed cell type and patient variability as the greatest driver of variance in the PBMC and CLL datasets, respectively (Additional file 1: Fig. S2b-d) and surpassing batch and donor for different cell types (Additional file 1: Fig. S2d-f). Although gene expression profiles varied notably, viable cells did not show signs of reduced RNA integrity across the time points (Additional file 1: Fig. S3).”*

*Modifications in the Methods:*

*“Variance analysis. To quantify and compare sampling time with other sources of variance, we followed two complementary approaches. First, we defined ρp as a universal measure of cell similarity, as Skinnider et al. [25] described this proportionality metric as the most accurate to measure cell-cell association. We then down-sampled T-cells and B-cells to 50 cells per sampling time to ensure that different cell types and time-points were equally represented. Likewise, we down-sampled each cluster of leukemic cells (one per donor) in a similar manner. We obtained a distance matrix by computing all pairwise ρp as described in Skinnider et al.. Finally, we sorted the distance matrix by cell type and time to allow for interpretation.*

*Second, we downsampled monocytes and NK cells as described before, and merged it with the T- and B-cell dataset. We then regressed the scran-normalized gene expression values of 5,282 genes (union of highly variable features of the merged and cell type-specific datasets) on one of four explanatory variables (cell type, time, donor and batch), and extracted a distribution of r2 values for each variable. To conduct a similar analysis in the Smart-seq2 dataset (T-cells) we used the plotExplanatoryVariables function from Scater [26].”*

4. The authors attempt to characterize the observed effect as a "cold-shock response" by computational means, but the analyses are not very convincing and no experimental validation is performed.

*Response: We fully agree with the Reviewer that the interpretation of the storage time-associated gene signature is overly simplistic. Although we detect clear markers of a cold-shock response (as highlighted by two master-regulators CIRBP and RBM3 in the top up-regulated genes in both PBMC and CLL datasets) and a global down-regulation of gene expression (more on that below), we now discuss the sampling effect also in a broader context. For instance, we detect general stress-related genes (JUN, JUNB, JUND, NFKBI) that were reported both as a consequence of ex vivo incubation (Baechler, Dvinge), but also due to collagenase digestion (van den Brink). It is important to note that the characterization of the sampling signature was not in the primary focus of the study, but rather the identification of such sampling artifact, its quantification and correction in future works.*

*“Further, two cold-shock master-regulators Cold Inducible RNA Binding Protein (CIRBP) and the RNA Binding Motif Protein 3 (RBM3), were amongst the top up-regulated genes in both datasets (Fig. 1f and Additional file 1: Fig. S6a). Comparing our profiles with a sampling time dependent signature detected by bulk gene expression analysis [11] and with tissue-dissociation signature [8], several bona-fide stress-regulators were consistently detected across signatures (NFKBI, JUN, JUND, JUNB; Additional file 1: Fig. S8a,b). However, there was a marginal global intersection (Additional file 1: Fig. S8a,c and Additional file 4: Table 3), which highlights the need for technology-specific benchmarking efforts of technical confounders. As an example, gene markers for splicing events detected in bulk analysis [12] were undetectable with scRNA-seq, whilst CIRBP and RBM3 were only found in single-cell experiments.”*

The authors write that they observe a "global downregulation of gene expression" (p3 l. 19) but it is not clear from the results presented if this is accurate, with only <200 downregulated genes, and a slight apparent reduction in average expression levels (please describe what the blue lines in Figure 1g and S6a indicate).

*Response: We agree with the Reviewer with the fact that a global down-regulation of gene expression is not consistent with the numbers provided. The reason for this low number of down-regulated genes is two-fold.*

*First, our main objective was to maximize the specificity of the differentially expressed genes (DEG) reported, reducing type I error at the expense of increased type II error (decreased statistical power). Thus, we only tested for differential expression for those genes with a minimum log (fold-change) of 0.25 (Seurat’s default value). However, in the context of a global down-regulation, it is expected that the vast majority of down-regulated genes will suffer a subtle decrease in gene expression, so we now also included them in the revised analysis.*

*Second, and in line with the previous point, we used a non-parametric statistical test (Wilcoxon test), which by definition has low statistical power. The choice for this method was motivated by previous benchmarking articles that showed the Wilcoxon test to be among the best-performing tools for scRNA-seq analysis (Soneson et al.). Moreover, Bieth et al. showed that with scran-normalized expression values (like ours), non scRNA-seq-tailored methods (like t-test or Wilcoxon) outperform tailored ones (MAST).*

*We now report 1,185 and 1,868 DEG that change their expression due to storage time (stringent multiple test correction and p-value cutoff) in the PBMC and CLL datasets, respectively. The majority of the DEG are down-regulated (73.16% in PBMC and 79.76% in CLL), shedding light to our main conclusion and in line with previous reports (Dvinge et al. and Baechler et al.). The global down-regulation also manifests in a significant reduction of the number of detected genes in time-biased samples, with the consequent increase in dropout rate (Supplementary Figure 6b). However, we still define the storage time-dependent signature with our initial approach to maximize the specificity of the effect in subsequent analysis.*

*“We detected 1,185 differentially expressed genes for PBMC (DEG, 318 up- and 867 down-regulated; Fig. 1f,g and Additional file 2: Table 1) and 1,868 for CLL samples (378 up- and 1,490 down-regulated; Additional file 1: Fig. S6a and Additional file 2: Table 1).”*

The authors mention of a similar effect being observed in a 2004 bulk transcriptomic study by Baechler et al. (ref. 5), however no direct comparison with the data from this study is performed. It is also unclear how the observed expression signature relates to previously reported “dissociation artifact” in scRNA-Seq data (Brink et al., ref. 9), which the authors cite later in a different context. Since the Brink et al. study involves scRNA-Seq data, and there appear to be some parallels in terms of genes induced (such as FOS and JUN), it would be important to perform a more detailed comparison with data from that study.

*Response: We agree with the Reviewer that a more thorough comparison with previous studies is necessary. Hence, we now compare our signature with the ones reported by Baechler et al. and van den Brink et al. (see Methods). We also cite a similar study by Dvinge et al., which performed a benchmarking of the effect of sampling time on leukemic transcriptomes measured by bulk RNA-seq. However, the latter is not included in the comparison, since the authors do not report p-values, and ranking by log2(fold-changes) gives a signature that is not consistent with their findings.*

*This comparison showed a high intersection in bona-fide stress-regulators (NFKBI, JUN, JUND, JUNB), but with a poor global overlap. This highlights the need to conduct technology-specific benchmarking efforts to find suitable markers for confounders. For instance, Dvinge et al. report 27 sampling time-dependent splicing events, which are not detectable by 3’ scRNA-seq methods. Likewise, we show the up-regulation of cold-shock regulators (CIRBP and RBM3) that were not detected by bulk transcriptomics approach.*

*“Comparing our profiles with a sampling time dependent signature detected by bulk gene expression analysis [11] and with tissue-dissociation signature [8], several bona-fide stress-regulators were consistently detected across signatures (NFKBI, JUN, JUND, JUNB; Additional file 1: Fig. S8a,b). However, there was a marginal global intersection (Additional file 1: Fig. S8a,c and Additional file 4: Table 3), which highlights the need for technology-specific benchmarking efforts of technical confounders. As an example, gene markers for splicing events detected in bulk analysis [12] were undetectable with scRNA-seq, whilst CIRBP and RBM3 were only found in single-cell experiments.”*

There are two issues with the gene ontology enrichment analysis: 1) the GO terms reported as enriched appear only indirectly related to the cold-shock response, 2) the authors show enrichment p-values of four GO terms for the PBMC and CLL datasets, respectively (Figure S6c,d). Are those GO terms the only ones with significant p-values? If not, it would be helpful to provide the full lists of significantly enriched GO terms, as a context for interpreting the results.

*Response: We agree with the Reviewer. We attribute the lack of specificity of the GO terms to the global downregulation, which is unspecific by definition. This is highlighted by a downregulation in housekeeping terms (i.e. actin nucleation) or cell type-specific functions (i.e. B-cell receptor signaling pathway); and is further strengthen by an up-regulation of terms like “negative regulation of translation” (PBMC) or “cellular response to oxygen levels” (CLL). Nevertheless, and consistent with our previous answer, we now broadened the interpretation of the DEG and GO terms. We also provide the full list of significant GO terms for PBMC, CLL and the four cell types (T-cells, NK, monocytes and B-cells) as new Supplementary Table 2.*

*Modification in the main text:*

*“Consistently, Gene Ontology (GO) enrichment analysis revealed a significant increase in the terms “negative regulation of translation” (PBMC) and “negative regulation of transcription by RNA polymerase II” (CLL) as well as a decrease in housekeeping functions such as actin nucleation (Additional file 1: Fig. S6c,d and Additional file 3: Table 2; p<0.001). In line, we detected a pronounced down-regulation of immune cell type specific genes (Fig. 1f, Additional file 1: Fig. S7a and Additional file 2: Table 1) and programs (Additional file 1: Fig. S7b,c and Additional file 3: Table 2) pointing to a loss of identity and function in prolonged storage conditions.”*

*Modification in the Methods:*

*“Integration of the sampling signatures. To compare the sampling time signatures detected for PBMC and CLL with other condition-specific signatures, we downloaded the DEG tables for the studies by Baechler et al. [11] and van den Brink et al. [8]. We defined as signature those genes with an adjusted p-value < 0.001. Furthermore, we subsetted the Baechler signature to the top 250 genes to harmonize the number of DEG across signatures. Finally, we calculated a signature-specific score using the AddModuleScore function from Seurat.”*

5. In the last part of the manuscript, the authors discuss strategies for addressing or avoiding the observed artifact which can be potentially very useful to the single cell community. While the authors propose a range of strategies, each one is studied and discussed in relatively little detail, and it ultimately unclear what the conclusions are of this part of the study and what bearing they may have for future study design. The authors correctly point out that regression-based approaches can be problematic, as they may accidentally remove signals of biological interest (p4, ll. 10-13). The authors also show that the artifact in PBMCs was greatly reduced after culturing for two days (Fig 2c,d), however they do not study how the culturing of primary immune cells affects their cell type composition and cell type-specific expression patterns. It is therefore unclear in what situations this approach would be useful.

*Response: We agree with the Reviewer with the fact that the effect of culturing and activating primary immune cells needs to be characterized in more detail. New analysis showed that cultured cells clustered separately from uncultured cells (new Supplementary Figure 11). Specifically, CD4+ T-cells showed an increase in cell cycle, which was the main biological process triggered upon incubation with anti-CD3 antibody (10.1186/s12864-019-5967-8). Differential expression analysis between cultured and uncultured cells revealed global increase in gene expression and the activation of multiple immune-related genes (new Supplementary Table 5). Culturing and activating PBMC removed monocytes while CD4+ T-cell count was proportionally increased (new Supplementary Figure 11). Thus, although culturing removed the sampling time artifact, it changed cell type composition and gene expression patterns. Nevertheless, for retrospective studies with unknown sampling time points, cell culturing presents a viable option to avoid artifacts. Further, auto-immune diseases are best modeled in vitro through immune cell activation, a treatment that simultaneously removes sampling effects.*

*“It is of note that culturing and activation results in specific gene expression profiles that allow the simulation of disease phenotypes (e.g. auto-immune diseases), but might also distort expression profiles observed in vivo (Additional file 1: Fig. S11 and Additional file 6: Table 5).”*

Finally, the authors follow up on a recent report that solid tissues can be effectively preserved at 4 degrees Celsius ("cold storage", Madissoon et al., ref. 15), and test for the presence of the artifact after cold storage. They show that this preservation appears to eliminate the expression artifact (Figure 2e,f). Out of all the strategies studied, this approach would appear to hold the most promise for practical applications, but unfortunately the authors only present only a cursory analysis of the data. For example, it would be important to analyze the expression profile of the genes that form the previously identified expression signature of the storage artifact.

*Response: We included two extra panels in the corresponding Supplementary Figure 12: 1) a differential expression analysis between PBMC stored 4ºC or RT before cryopreservation and 2) a comparison of the number of DEG across sampling times between storage at 4ºC and at 21ºC in CLL samples. The latter suggest that the magnitude of storing cells at 4ºC is very small as compared to sample stored at 21ºC.*

*“Although we detected an up-regulation of stress-regulators (NFKBIA, FOS, JUN, JUNB) in PBMC stored >24h at 4ºC (Additional file 1: Fig. S12c), we identified between 254 (2h) and 362 (24h) DEG in CLL cells, notably less than when kept at RT (between 797 and 1,956 DEG at 2h and 24h, respectively; Additional file 1: Fig. S12d)”*

Furthermore, it is not clear how these experiments were designed, as the manuscript refers to cells "stored at 4°C (24h/48h)". Which cells were stored for 24h and which for 48h, and whether they were combined for analysis purposes only, or immediately after cell isolation, does not seem to be indicated anywhere.

*Response: This is now better clarified in the main text.*

*“Indeed, when PBMC or CLL samples were stored at 4ºC until cryopreservation (24 and 48 h for PBMC; 2, 4, 6, 8 and 24h for CLL), we did not detect global gene expression artifacts; an effect observed for both healthy and CLL samples and replicated across donors and technologies (Fig. 2e,f and Additional file 1: Fig. S12a,b).”*

Minor comments:

1. Basic metrics relating to the experiments are missing, in particular, the number of cells obtained for each time point (before and after QC), and the number of cells belonging to each cell type. Maybe these could be provided in a supplementary table.

*Response: We quantified the number of cells before after QC for every time-point, temperature and donor in both experiments (PBMC and CLL). Moreover, we also provide the number of cells per cell type in both PBMC and CLL. All this information is now provided as Supplementary Table 6.*

2. How do these numbers/ fractions of cell-types in PBMC compare to previous studies using single cell genomics, flow cytometry, etc.? Do the authors have any comments on inter-individual variability in terms of number fractions of each cell-type in PBMC? Or are such observations confounded by technical challenges and cannot be made with confidence?

*Response: Although the cell encapsulation into droplets could introduce a composition bias of cell types and states, we expect that this effect is minor for the analysis of PBMC. Following Ficoll isolation, there are no major differences in cell sizes and cell viability making a biased cell capture unlikely. The compositions reported in our study are highly comparable to those observed in previous works, such as Zheng et al. performing scRNA-seq for 250,000 mostly PBMC samples.*

Comments for Figure 1:

a. How many cells in each condition for Figures a,b,d?

b. While the visualizations in panels a/b/d broadly convey the main results described in the paper, some aspects are problematic:

i. In panels a and b, cell diameters are very large so that most cells are probably completely occluded from the reader's view. This could be improved by redrawing the plots with a smaller cell diameter, as in panel d.

ii. Also, it appears as if cells from certain time points are occluded behind cells from other time points (e.g., very few cells from the 2h time point are visible in a and likely occluded behind cells from the 0h time point). If this is the case, this could be improved by randomizing the order in which cells are drawn (depending on the visualization tool used, this may be technically challenging - in this case reducing the cell diameter should at least alleviate the problem; see above).

iii. Since color is used to indicate the time point, the cell type assignments are not clear. This could be improved by manually drawing boundaries around each cluster. If clusters do not cleanly separate by location in the t-SNE plot, an approximate boundary could be indicated with a dashed line to improve the readability of the figure.

c. It is unclear whether panel c shows results from a single (global) PCA, for cells grouped by cell type, or whether a PCA was conducted for each cell type separately. This could be clarified in the figure legend.

d. For panel e, it is unclear what the "p-value in z-score scale" means. If the values represent z-scores, the label of the y-axis should simply read "z-score".

e. In panel e, significance thresholds based on p-values are displayed, but it is not clear what statistical test was used to obtain those p-values. This information should be included in the figure legend.

f. In reference to panel e and Figure S4, the claim is made that "Specifically, we detected reduced expression for genes that lose open chromatin sites, a trend that was more pronounced analyzing enhancers compared to promoter sites [...] and suggesting that specific enhancer sets trigger the response to temperature changes." (p3 ll. 1-4) However the authors do not quantify this effect or test its significance, and apparently the claim is based simply on a visual comparison between the violin plots, which is quite subjective. It would therefore seem appropriate to qualify this claim, provide additional evidence in the form of quantitative analyses, or omit this claim from the manuscript as it does not appear to be a central result.

*Response: We thank the Reviewer for the attention to detail. All the changes have been applied in the corresponding panel or figure legend.*

In summary, the study focuses on an important question related to blood sample preparation for scRNA-Seq analysis, and the experimental and computational methodologies applied appear technically sound. However, it is unclear exactly what the implications of the presented results are for the design of future scRNA-Seq studies. A discussion on the generalizability of the findings and methods suggested to bypass the technical challenges to other systems will be helpful.

*Response: We thank the Reviewer for the positive evaluation of our study. We added the following discussion as note of caution that the here detected gene signatures could be immune cell specific and that dedicated benchmarking efforts are required for other sample types.*

*“However, there was a marginal global intersection (Additional file 1: Fig. S8a,c and Additional file 4: Table 3), which highlights the need for technology-specific benchmarking efforts of technical confounders.”*

*“Despite the substantial impact on single-cell datasets, the here proposed computational corrections, cell culture and storage adjustments allow the design of reliable retro- or prospective studies of immune cells from archived sample collections. However, sampling effects can be tissue and cell type specific, thus, dedicated benchmarking efforts are required for sample types others than the here tested.”*

**Second round of review**

**Reviewer 2**

None
